# Effect of a new formulation of micronized and ultramicronized N-palmitoylethanolamine in a tibia fracture mouse model of complex regional pain syndrome

**DOI:** 10.1371/journal.pone.0178553

**Published:** 2017-06-08

**Authors:** Roberta Fusco, Enrico Gugliandolo, Michela Campolo, Maurizio Evangelista, Rosanna Di Paola, Salvatore Cuzzocrea

**Affiliations:** 1 Department of Chemical, Biological, Pharmaceutical and Environmental Sciences, University of Messina, Messina, Italy; 2 Institute of Anaesthesiology and Reanimation, Catholic University of the Sacred Heart, Rome, Italy; 3 Department of Pharmacological and Physiological Science, Saint Louis University School of Medicine, St. Louis, Missouri, United States of America; Universita degli Studi di Napoli Federico II, ITALY

## Abstract

Complex regional pain syndrome type 1 (CRPS-I) is a disabling and frequently chronic condition. It involves the extremities and is a frequent consequence of distal tibia and radius fractures. The inflamed appearance of the affected CRPS-I limb suggests that local production of inflammatory mediators may be implicated in the ensuing etiology. A rodent tibia fracture model, characterized by inflammation, chronic unilateral hindlimb warmth, edema, protein extravasation, allodynia and hyperalgesia resembles the clinical features of patients with acute CRPS-I. N-palmitoylethanolamine (PEA), a member of the family of naturally-occurring N-acylethanolamines, is well-known for its ability to modulate inflammatory processes and regulate pain sensitivity. However, the large particle size and lipidic nature of PEA may limit its bioavailability and solubility when given orally. Micronized formulations are frequently used to enhance the dissolution rate of drug and reduce its variability of absorption when orally administered. The aim of this study was to assess the effects of a formulation of micronized and ultramicronized PEA (PEA-MPS), given orally in a mouse model of CRPS-I. CD-1 male mice were subjected to distal tibia fracture and divided into two groups: control and treated with PEA-MPS (PEA micronized 300 mg/kg and ultramicronized 600 mg/kg). Sensibility to pain was monitored in all mice throughout the course of the experiment. Twenty-eight days after tibia fracture induction animals were sacrificed and biochemical parameters evaluated. The PEA-MPS-treated group showed an improved healing process, fracture recovery and fibrosis score. PEA-MPS administration decreased mast cell density, nerve growth factor, matrix metalloproteinase 9 and cytokine expression. This treatment also reduced (poly-ADP)ribose polymerase activation, peroxynitrite formation and apoptosis. Our results suggest that PEA-MPS may be a new therapeutic strategy in the treatment of CRPS-I.

## Introduction

Algodystrophy, or Complex Regional Pain Syndrome type I (CRPS-I), is a painful syndrome characterized by vasomotor and sensory disturbances, edema and functional impairment. The first report of Algodystrophy was made by the German surgeon Paul Sundeck more than 110 years ago. He described the case of a patient suffering from “acute inflammatory bone atrophy” with the accepted clinical signs of inflammation (*functio laesa*, *dolor*, *tumor*, *rubor*, and *calor*) in association with a “patchy” osteoporosis [1]. Even today, a full understanding of the pathophysiological mechanisms underlying CRPS-I remains elusive. The most convincing pathogenic hypothesis is a local process of neuroinflammation, perhaps associated with clinical symptoms occurring in the first stage of the disease (eritrosis, edema, sweating and increased local temperature) followed later by microvascular damage and microcirculation impairment observed in most patients (“dystrophic” or “cold” phase) with reduced edema, decreased local temperature and presence of subcianosis [2].

Neuroinflammation plays an important role in the pathogenesis of both peripheral and central chronic pain [3, 4]. Resolution of inflammation is controlled by the elaboration of soluble products [5].Among these numerous lipidic signaling molecules whose role is to restore tissue homeostasis by suppressing the inflammatory process and by regulating pain sensitivity through moderating the flow of nociceptive signs to the central nervous system [6]. The N-acylethanolamines comprise one such family of molecules, whose main members are N-arachidonoylethanolamine (anandamide) and its congeners N-oleoylethanolamine, N-stearoylethanolamine and N-palmitoylethanolamine (PEA) [7]. Animal studies demonstrating the capability of PEA to modulate pain and inflammation propose that this endogenous fatty acid amide is part of a complex homeostatic system driving the basal threshold of both pain and inflammation. PEA anti-inflammatory activity has been amply shown in numerous animal models of inflammation, such as adjuvant-induced arthritis, ischemia reperfusion injury, idiopatic pulmonary fibrosis, carrageenan-induced paw edema and tuberculin hypersensitivity [8, 9]. PEA, as an endogenous compound, has a double therapeutic effect (that is, anti-nociceptive and anti-inflammatory) without adverse effects at pharmacologically relevant doses [10, 11]. Recent report highlights a new PEA potential mechanism of action mediated by the induction of the CB2 up-regulation [12]. The large particle size and lipidic nature of PEA limit its solubility and bioavailability. Reports in the literature also highlighted the capability of other PEA formulations in the possible treatment of abnormal pain induced by several experimental models [13–17]. The micronization technique can be used to achieve microparticles <10 μm) [18, 19], with increased surface area and rate of dissolution [20], together with a reduced variability of absorption [21]. In this study, we investigated the effect of oral administration of a formulation of micronized and ultramicronized PEA (PEA-MPS) in a model of CRPS-I.

## Materials and methods

### Animals

Male adult CD1 mice (25–30 g, Harlan, Italy) were placed in a controlled location and provided with standard rodent water and chow *ad libitum*. Mice were accommodated in stainless steel cages in a room kept at 22 ± 1°C with a 12-h dark/light cycles. The animals were familiarized to their setting for one week. The study was approved by the University of Messina Review Board for the care of animals. All animal experiments complied with regulations in USA, Europe and Italy. All the experiments followed the ARRIVE guidelines.

### Fracture surgery

On day 0, mice were sedated with isoflurane, the right hindlimb enveloped in stockinet (2.5 cm wide) and the distal tibia fractured using a pair of pliers (Visegrip, Petersen Manufacturing) modified with a 3-point jaw. The hindlimb was then covered in casting tape (Delta-Lite, Johnson & Johnson) to allow the hip, ankle and knee to flex. The cast stretched from the metatarsals to a spica shaped around the abdomen. The cast over the paw only reached to the plantar surface; a space was left over the dorsum of the paw and ankle to avoid constriction when edema developed post-fracture. The cast was covered in galvanized wire mesh to avoid chewing by the animal. Mice were given buprenorphine (0.03 mg/kg, subcutaneous) and saline immediately after surgery. At 28 days the mice were anesthetized with isoflurane and the cast eliminated [22].

### Experimental groups

Mice were randomly divided into the following groups (n = 10 for each group):

- Sham + vehicle group: vehicle solution (*carboxymethylcellulose 1*.*5% wt/vol in saline)* was administered orally for 28 days.
- Sham + PEA-MPS group: mice were treated orally daily with PEA-MPS (300 mg/kg PEAm and 600 mg/kg PEA-um®) for 28 days.
- Fracture + vehicle group: vehicle solution (*carboxymethylcellulose 1*.*5% wt/vol in saline)* was administered orally daily 1 h after surgery for 28 days.
- Fracture + PEA-MPS group: mice were treated orally daily with PEA-MPS (300 mg/kg PEAm and 600 mg/Kg PEA-um®) 1 h after surgery for 28 days.

The dose of PEA-MPS was chosen based on previous experiments [23].

In this study, we have demonstrated the beneficial effects of PEA in reducing edema formation and thermal hyperalgesia in carrageenan-induced inflammation in the rat paw. These results show the differential effects exerted on the degree of inflammation by micronized PEA-m and ultramicronized PEA-um, vs nonmicronized PeaPure.

We have done the calculation to know the dose of PEA-MPS that should be assumed by human. According to the formula applied for the conversion from animal to human, the dose of PEA-MPS that should be taken would be about 170,26 mg/Kg (56,75 mg/Kg PEAm and 113,51 mg/Kg PEA-um®), reasonably low [24]. Human Equivalent Dose PEA-MPS (mg/kg) = (300x[7/37]) + (600x[7/37]) = 170,26 mg/Kg.

The minimum number of animals for each group was calculated using the statistical test a priori power analyzes of the G-power software. This statistical test provides an efficient method for determining the sample size necessary to perform the experiment before the experiment the same is actually conducted.

### Behavioral measurements

Three tests were employed to calculate pain behavior: mechanical nociception measured by the withdrawal response to von Frey filament application, thermal nociception measured by the withdrawal response to thermal stimulus (hot plate test), and subjective pain assessed using a pain rating scale [25].

#### Mechanical nociception

To measure mechanical allodynia in the rats an up–down von Frey testing paradigm was used. Mice were located beneath a clear plastic cylinder (20 cm in diameter) on an elevated mesh floor and acclimatated for 15 min. Withdrawal responses to mechanical stimulation were assessed using calibrated von Frey filaments placed from underneath the chamber through openings in the mesh floor against approximately the middle of the hind paw plantar skin at the fractured side. The fibers were presented according to the up–down method of Dixon to generate six responses in the immediate vicinity of the 50% threshold. The filament was pressed until it slightly curved and then it was left in that position for 6 s. Each filament was applied once, beginning with 0.008 g and continuing until a withdrawal response was considered positive. After a pause of 5–10 min, each filament was again used once, beginning with 0.008 g until a withdrawal response was achieved. This was replicated a third time 5–10 min later. The withdrawal threshold was considered the lowest force producing a response from the three tests [26].

#### Thermal nociception

Thermal nociception was assessed by a modified hot plate test [27]. Thermal nociception (thermal latency) was reflected by the time that a mouse would leave its hind paw on a hot plate at 52°C. The paw was shifted from the plate by the investigator after a maximal time of 12 s to avoid thermal hyperalgesia and injury. This test was replicated three times.

#### Subjective pain scale

A subjective pain rating scale (0–5) was utilized to quantify the pain. Zero is normal, 1 is crimping of the toes, 2 is evasion of the paw, 3 is limited weight bearing, 4 is non–weight bearing, and 5 is evasion of any contact with the hind limb.

### Radiographic analysis

Lateral radiographs of the tibiae from mice were taken using a X-ray apparatus (FX Pro Brucher, Italy). Mineralized callus realization and bony association at the fracture site were evaluated. Callus total volume (TV) assessed the volume of recently formed tissues and low-density bone volume (BVl) assessed the volume of recently formed mineralized tissue in callus. BV1 and TV were both calculated by a decrease of the volume in fractured tibia with the volume of the contralateral bone.

### Histological analysis

On day 28 after fracture, mice were sacrificed by anaesthetic overdose. Tibiae were collected and post-fixed in 10% formalin and decalcified in EDTA for 24 h. The specimens were then embedded in paraffin, 5 μm sections prepared, stained with hematoxylin/eosin, and analysed by histomorphometry [28–30]. Contrast and illumination were established by examining the most intensely labelled pixels and applying backgrounds that allowed clear image of structural details while keeping the highest pixel intensities close to 200. The same backgrounds were used for all images acquired from the other samples that had been managed in parallel. Digital images were collected and figure montages arranged using Adobe Photoshop CS6 (Adobe Systems; Milan Italy).

### Masson trichrome, safranin O/fast green and toluidine blue staining

The degree of fibrosis was evaluated according to the manufacturer’s protocol (Bio-Optica, Italy, Milan), with tissue sections being stained with Masson trichrome. In order to evaluate the presence of osteoclasts and osteoblasts, samples were stained with safranin O/fast green, again according to the manufacturer’s protocol (Bio-Optica, Italy, Milan). Tissue sections were also stained with toluidine blue to evaluate the number and degranulation of mast cells, together with histopathological analysis of the callus and cartilage volumes. Sections were deparaffinised in xylene and dehydrated by a graded series of ethanols, 4 min in each. The sections were next placed in water for 5 min, transferred to toluidine blue for 4 min and then blotted carefully. Sections were next placed in absolute alcohol for 1 min, then in xylene, followed by mounting on a glass slide using Eukitt (Bio-Optica, Italy, Milan). Cartilage was stained blue with mast cells coloured purple. The mast cell count and cartilage analysis were performed on each slide using an Axiovision Zeiss (Milan, Italy) microscope.

### Immunohistochemical localization of nerve growth factor (NGF), matrix metalloproteinase (MMP)9, tumor necrosis factor alpha (TNF-α), interleukin-1beta (IL-1β), nitrotyrosine, poly(ADP-ribose) (PAR), Bax and Bcl-2

At 28 days post-surgery, tibia fractures were fixed in PBS-buffered formaldehyde 10% (w/v) and embedded in paraffin. Seven μM sections from each tissue were prepared. After deparaffinization, endogenous peroxidase was quenched with 0.3% (v/v) hydrogen peroxide/60% water for 30 min. Tissue was permeabilized with 0.1% (w/v) Triton X-100 in PBS for 20 min. Slides were incubated in 2% normal goat serum in PBS to block non-specific binding. Endogenous avidin and biotin binding sites were blocked vy sequential incubation with avidin and biotin (Vector Laboratories, Burlingame, CA) for 15 min. Sections were then incubated overnight with one of the following primary antibodies: anti-NGF (E-12: sc-365944, 1:460 in PBS, Santa Cruz Biotechnology), anti-MMP9 (C-20: sc-6840, 1:360 in PBS, Santa Cruz Biotechnology), anti-TNF-α (H-156: sc-8301, 1:460 in PBS, Santa Cruz Biotechnology), anti-IL-1β (H-153: sc-7884, 1:460 in PBS, Santa Cruz Biotechnology), anti-nitrotyrosine (1:460 in PBS, Millipore, 06–284), anti-PAR (H-250: sc-7150, 1:560 in PBS, Santa Cruz Biotechnology), anti-Bax(P-19: sc-526, 1:460 in PBS, Santa Cruz Biotechnology) or anti-Bcl-2 (N-19: sc-492, 1:360 in PBS, Santa Cruz Biotechnology). Sections were washed with PBS and incubated with secondary antibody. Specific labeling was visualized with a biotin-conjugated goat anti-rabbit IgG and avidin–biotin peroxidase complex (Vector Laboratories, Burlingame, CA). Immunohistochemical images were taken using a Zeiss microscope and Axio Vision software. For graphic display of densitometric analyses, the intensity of positive staining (brown staining) was measured by computer-assisted color image analysis (Leica QWin V3, UK). The percentage area of immunoreactivity (determined by the number of positive pixels) was expressed as a percent of total tissue area (red staining). Contrast and illumination were established by examining the most intensely labelled pixels and applying backgrounds that allowed clear image of structural details while keeping the highest pixel intensities close to 200. The same backgrounds were used for all images acquired from the other samples that had been managed in parallel. Digital images were collected and figure montages arranged using Adobe Photoshop CS6 (Adobe Systems; Milan Italy).

### Materials

All compounds were purchase from Sigma-Aldrich (Milan, Italy). All chemicals were of the highest grade available. All stock solutions were prepared in non-pyrogenic saline (0.9% NaCl; Baxter, Italy). PEA-MPS was kindly provided by Epitech Group SpA (Saccolongo, Italy).

### Statistical analysis

All values are represented as mean±standard error of the mean (SEM) with ‘n’ representing the number of experimental observations per cohort. In experiments involving histology, the figures shown are representative of at least three experiments performed on different days. The results were analyzed by one-way ANOVA followed by a Bonferroni *post-hoc* test for multiple comparisons. All statistical analyses were carried out using GraphPad Prism® Version 5.00 statistical software. A p-value less than 0.05 was considered significant. *p<0.05 vs. sham, ° *p*<0.05 vs vehicle.

## Results

### Effect of PEA-MPS treatment on fracture-induced nociceptive behavior

**Fig 1:**
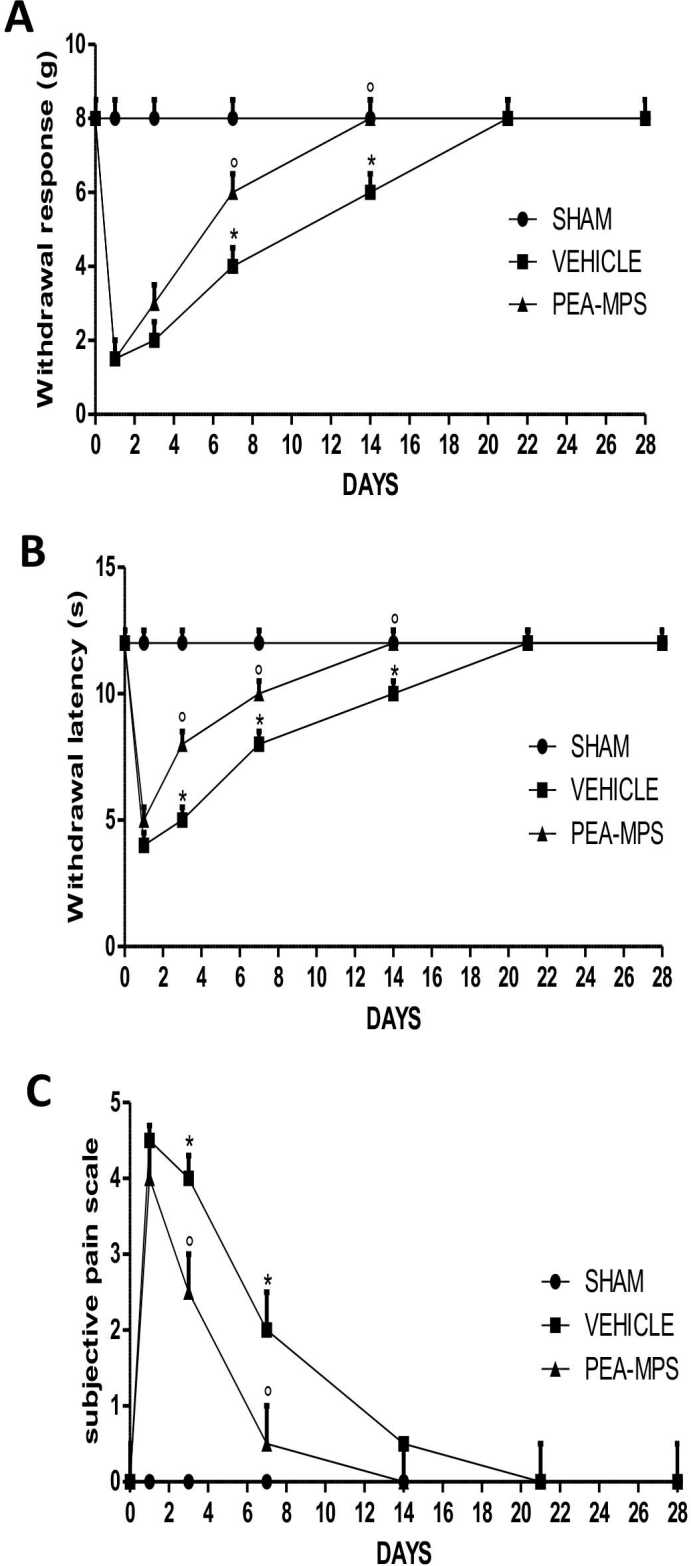
Efficacy of PEA-MPS on fracture-induced nociceptive behaviour. Twenty-eight days after fracture three tests were used to assess pain behaviour. (A) Withdrawal response to von Frey filaments-induced mechanical hyperalgesia in the fractured hind paw. PEA-MPS treatment increased the withdrawal response. (B) Thermal nociception was assessed by a modified hot plate test. PEA-MPS treated-mice showed increased withdrawal latency compared to vehicle treated-mice. (C) Subjective pain scale evaluating pain in the fractured hind paw. PEA-MPS treatment decreased pain compared to vehicle treated-mice. A p-value less than 0.05 was considered significant. *p<0.05 vs. sham, ° *p*<0.05 vs vehicle.

Mechanical nociception, thermal nociception and subjective pain were significantly modified by treatment with PEA-MPS as compared to the vehicle-treated group. Mechanical hyperalgesia and allodynia were calculated at the hind paw by score values measured from the von Frey filament test. As shown in Fig 1A, withdrawal response to mechanical stimulus increased in PEA-MPS treated-mice, indicating a decreased nociception, compared to vehicle-treated mice. The same pattern was observed in the hot plate test for thermal nociception (Fig 1B), with response latency in vehicle treated-mice showing an increased nociception that was significantly reduced in PEA-MPS-treated mice in compared to sham mice. The subjective pain scale (Fig 1C) was significantly decreased in PEA-MPS treated-mice compared to vehicle treated-mice, while sham mice remained unchanged.

### Effect of PEA-MPS treatment on fracture healing process

**Fig 2:**
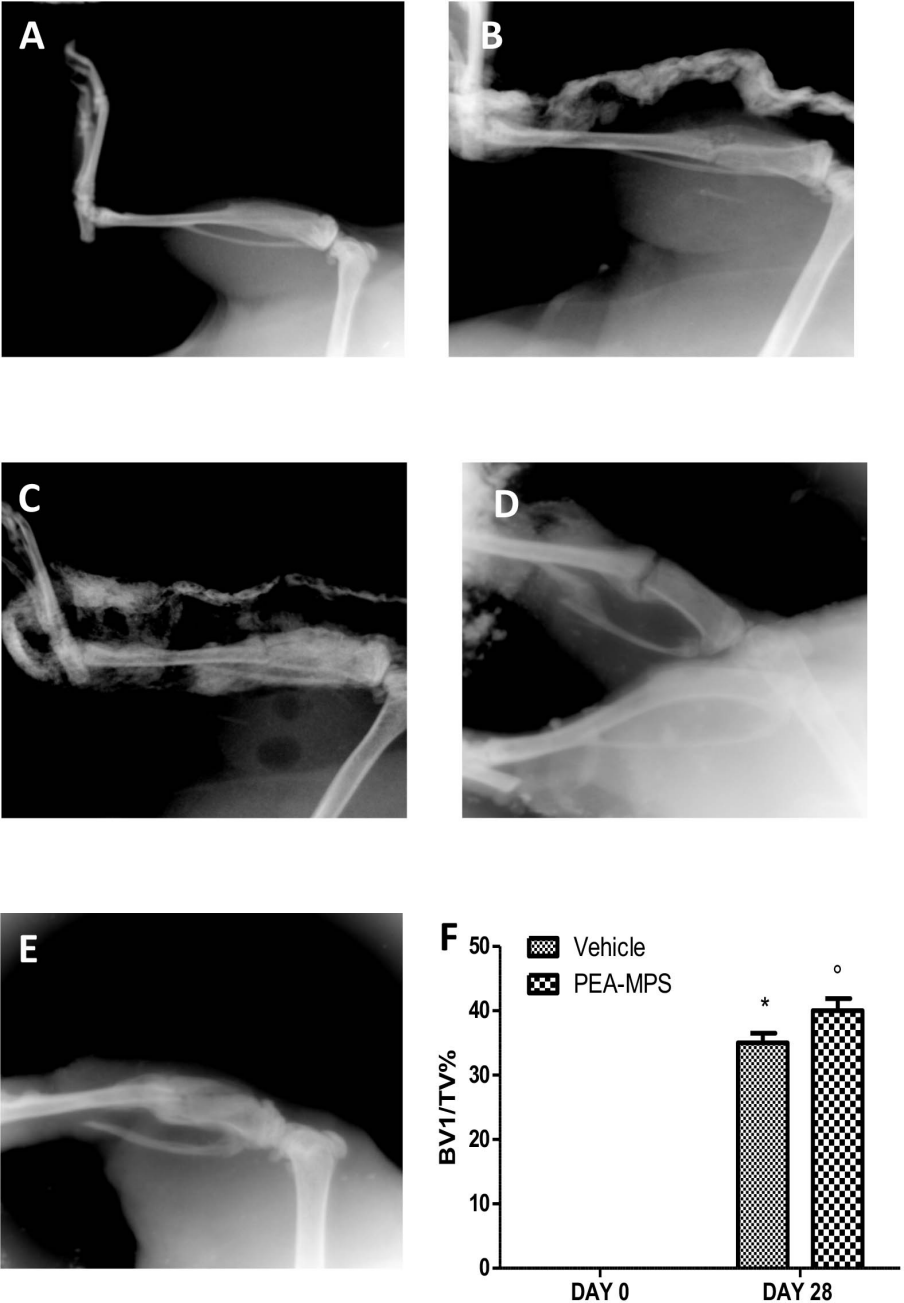
Efficacy of PEA-MPS on fracture healing process. Mice with tibia fracture were treated orally with vehicle (B) or PEA-MPS (C). Twenty-eight days later PEA-MPS treatment accelerated the fracture healing process (E) compared to vehicle-treated mice (D). PEA-MPS also stimulated callus bridging and increased bone density (F). A p-value less than 0.05 was considered significant. *p<0.05 vs. sham, ° *p*<0.05 vs vehicle.

To verify correct fracture execution and to determine the effect of PEA-MPS treatment on fracture repair, mice were subjected to X-ray analysis. No difference between vehicle-treated mice (Fig 2B and 2F) and PEA-MPS-treated mice (Fig 2C and 2F) was noted immediately after the fracture (day 0). At 28 days post-surgery the callus area was significantly larger in PEA-MPS-treated mice (Fig 2E and 2F) compared to vehicle-treated animals (Fig 2D and 2F).

### Effect of PEA-MPS treatment on facture recovery and fibrosis

**Fig 3:**
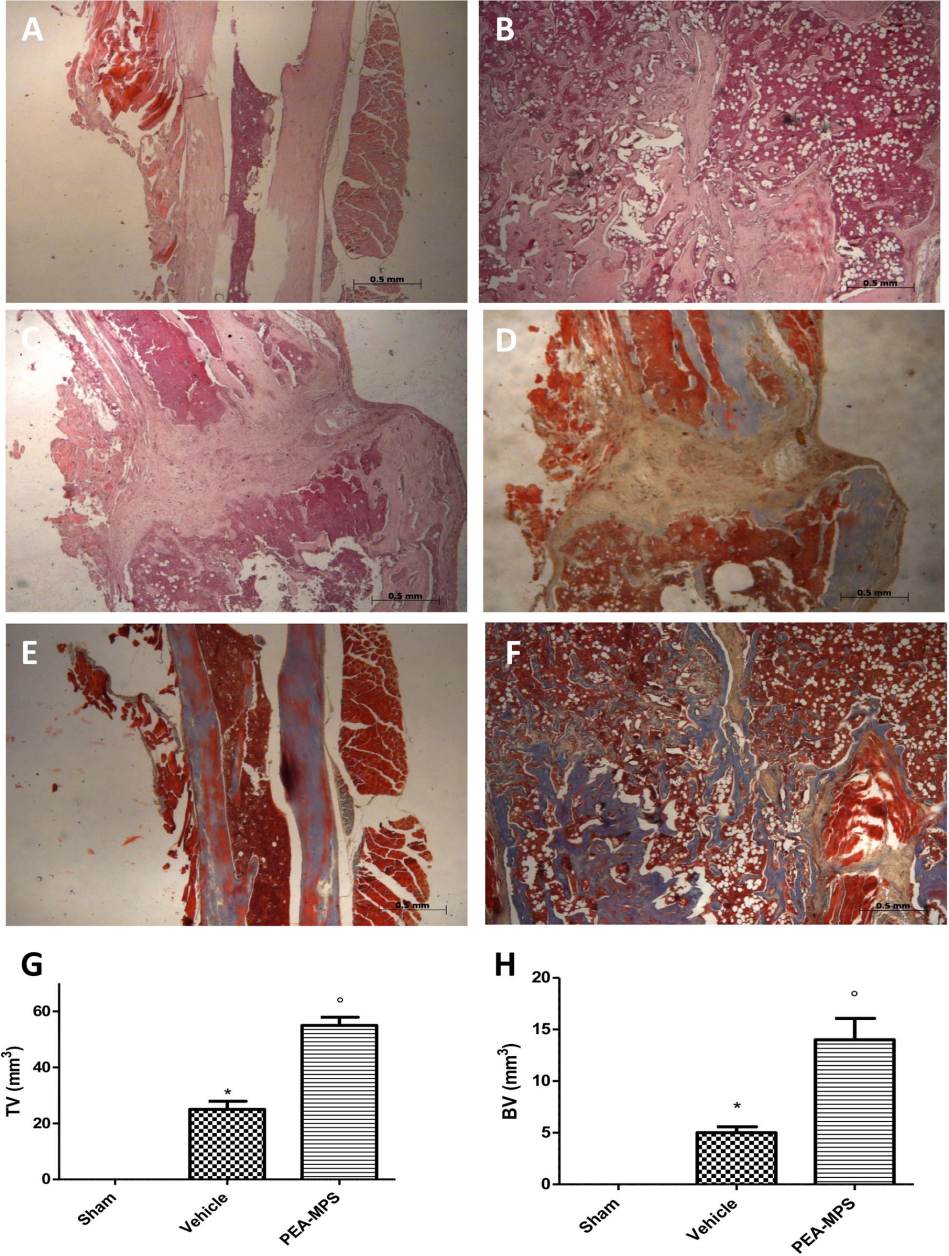
Efficacy of PEA-MPS on facture recovery and fibrosis. Twenty-eight days after fracture histological examination showed more callus formation and new woven bone in PEA-MPS-treated mice (B and G, and Figure b in S1 File) compared to vehicle-treated animals (C and G, and Figure c in S1 File). Masson trichrome staining demonstrated an increased fibrotic area in the PEA-MPS group (F and H, and Figure f in S1 File) compared to the vehicle group (E and H, and Figure e in S1 File). A p-value less than 0.05 was considered significant. *p<0.05 vs. sham, ° *p*<0.05 vs vehicle.

Twenty-eight days after surgery mice were sacrificed and longitudinal sections of tibia stained with hematoxylin/eosin. Tissues from PEA-MPS-treated mice (Fig 3B and 3G) showed more callus formation and new woven bone with respect to vehicle-treated mice (Fig 3C and 3G). At this time the degree of fibrosis, assessed by Masson trichrome staining, demonstrated a blue fibrotic area that was larger in the PEA-MPS group (Fig 3F and 3H) compared to the vehicle group (Fig 3E and 3H).

### Effect of PEA-MPS treatment on mast cell density and NGF expression

**Fig 4:**
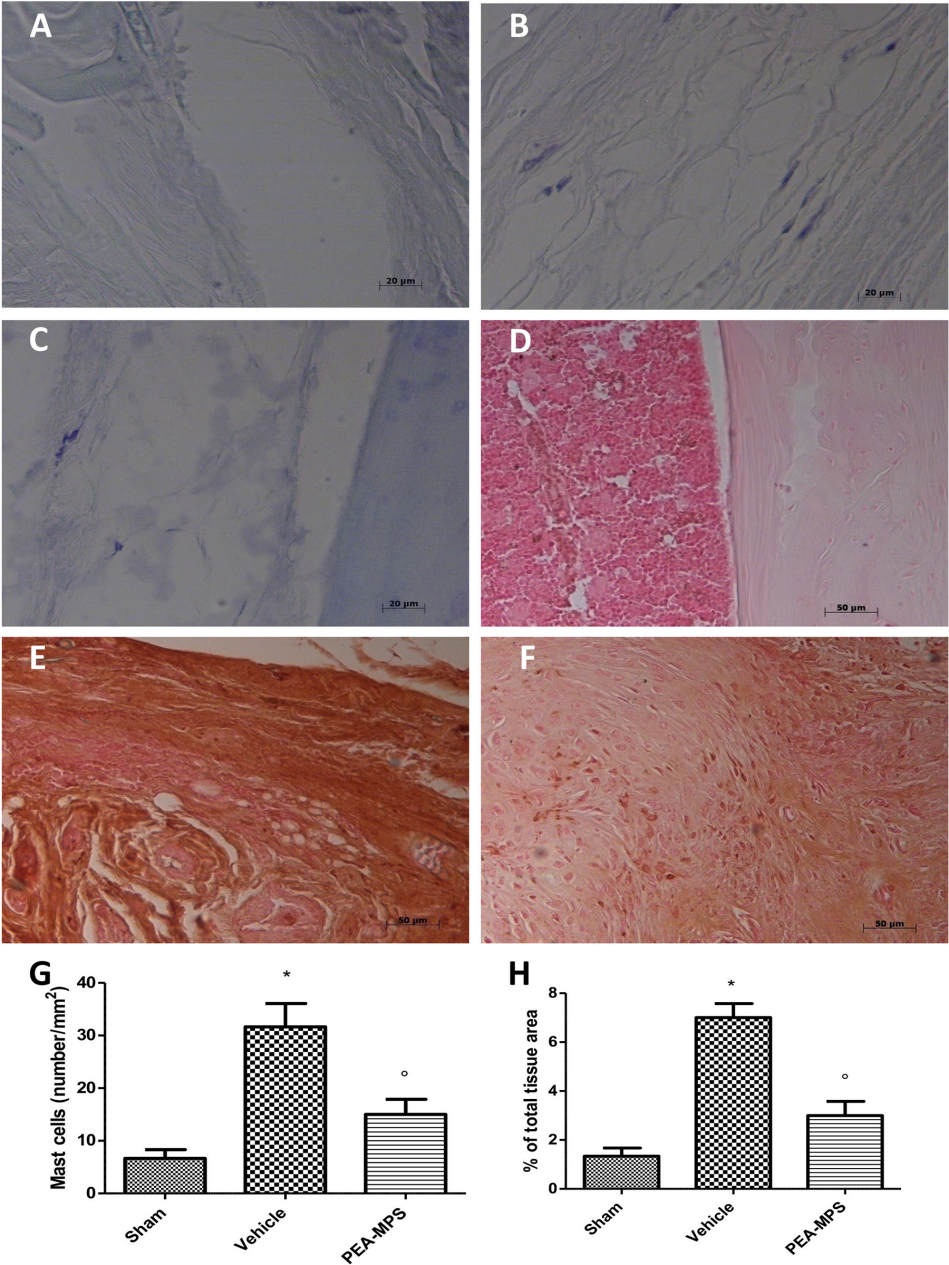
Efficacy of PEA-MPS on mast cell density and NGF expression. Mast cell number was increased in vehicle-treated mice (B and G, and Figure b in S2 File) compared to the sham group (A and G, and Figure a in S2 File). PEA-MPS treatment significantly reduced the tibia fracture-induced increase in mast cell number (B and G, and Figure b in S2 File). Tissue from vehicle-treated mice showed an increased NGF immunostaining (E and H, and Figure e in S2 File) compared to sham-treated mice (D and H, and Figure d in S2 File) which was reduced by PEA-MPS treatment (F and H, and Figure f in S2 File). A p-value less than 0.05 was considered significant. *p<0.05 vs. sham, ° *p*<0.05 vs vehicle.

Toluidine blue staining was used to assess mast cell number. Mast cell number was increased following distal tibia fracture in vehicle-treated mice (Fig 4B and 4G) compared to sham animals (Fig 4A and 4G). In contrast, PEA-MPS treatment significantly reduced mast cell number (Fig 4B and 4G). Immunohistochemical analysis of tissues collected from vehicle-treated mice showed an increase of NGF staining (Fig 4E and 4H) which was lower in PEA-MPS-treated mice (Fig 4F and 4H). Sham-treated mice did not show any significant NGF immnostaining (Fig 4D and 4H).

### Effect of PEA-MPS treatment on MMP9 expression and cartilage formation

**Fig 5:**
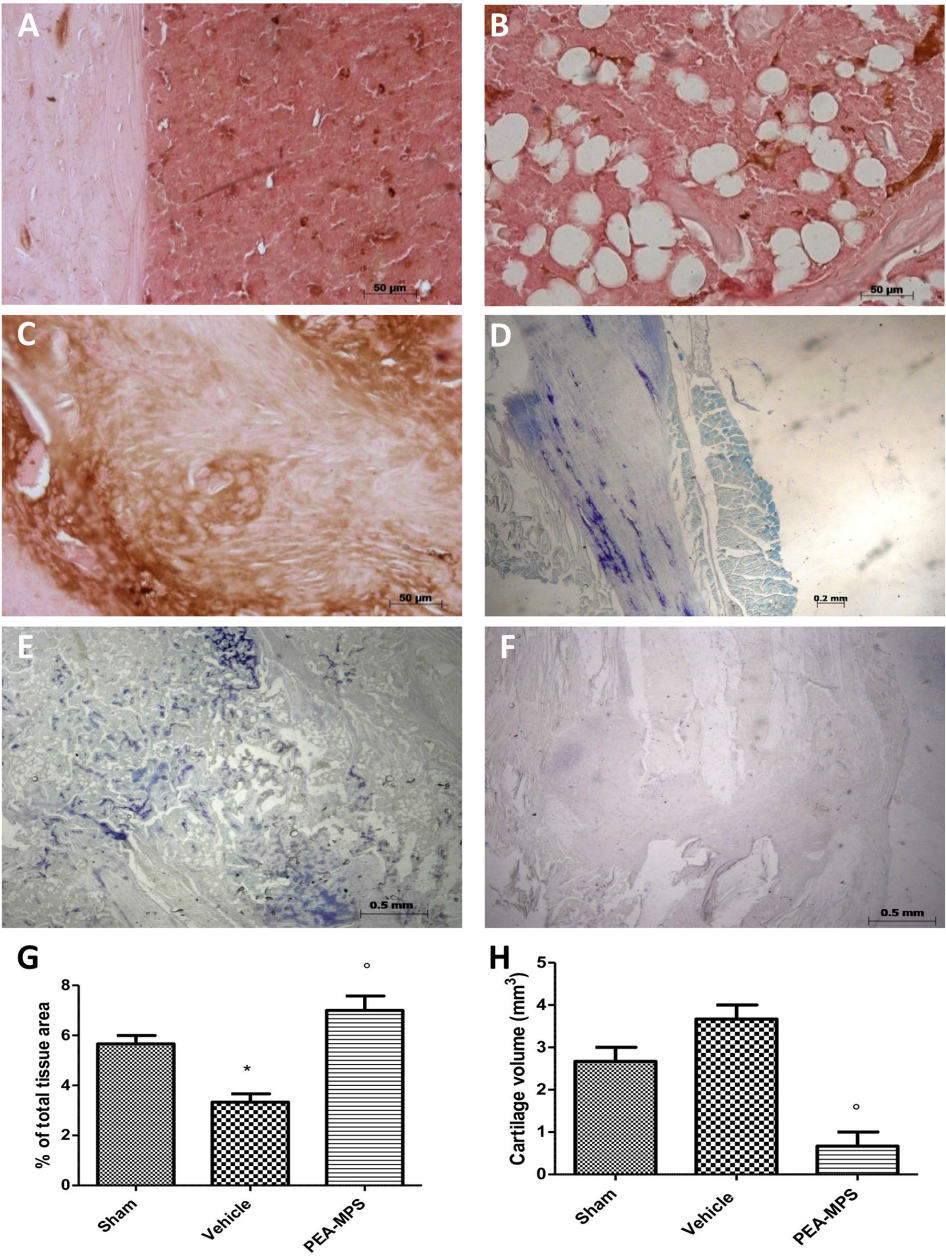
Efficacy of PEA-MPS on MMP9 expression and cartilage formation. MMP9 expression was analyzed immunohistochemically 28 days after surgery. PEA-MPS-treated mice showed an increased staining (C and G, and Figure c in S3 File) compared to vehicle-treated mice (B and G), while tissue from sham-treated mice showed basal MMP9 expression (A and G, and Figure a in S3 File). Toluidine blue staining demonstrated a significantly greater amount of cartilage area in PEA-MPS-treated mice (F and H, and Figure f in S3 File) compared to the vehicle group (E and G, and Figure e in S3 File). A p-value less than 0.05 was considered significant. *p<0.05 vs. sham, ° *p*<0.05 vs vehicle.

Twenty-eight days after surgery MMP9 expression was analysed in tissues collected from sham, vehicle and PEA-MPS-treated mice. MMP9 expression was up-regulated in PEA-MPS-treated mice (Fig 5C and 5G), compared to vehicle-treated mice (Fig 5B and 5G). Sham-treated mice showed a basal expression of MMP9 (Fig 5A and 5G). PEA-MPS-treated mice had a significantly lower amount of cartilage area (Fig 5F and 5H) as assessed by toluidine blue staining, compared to vehicle-treated mice (Fig 5E and 5G).

### Effect of PEA-MPS treatment on osteoclasts on tissue sections

**Fig 6:**
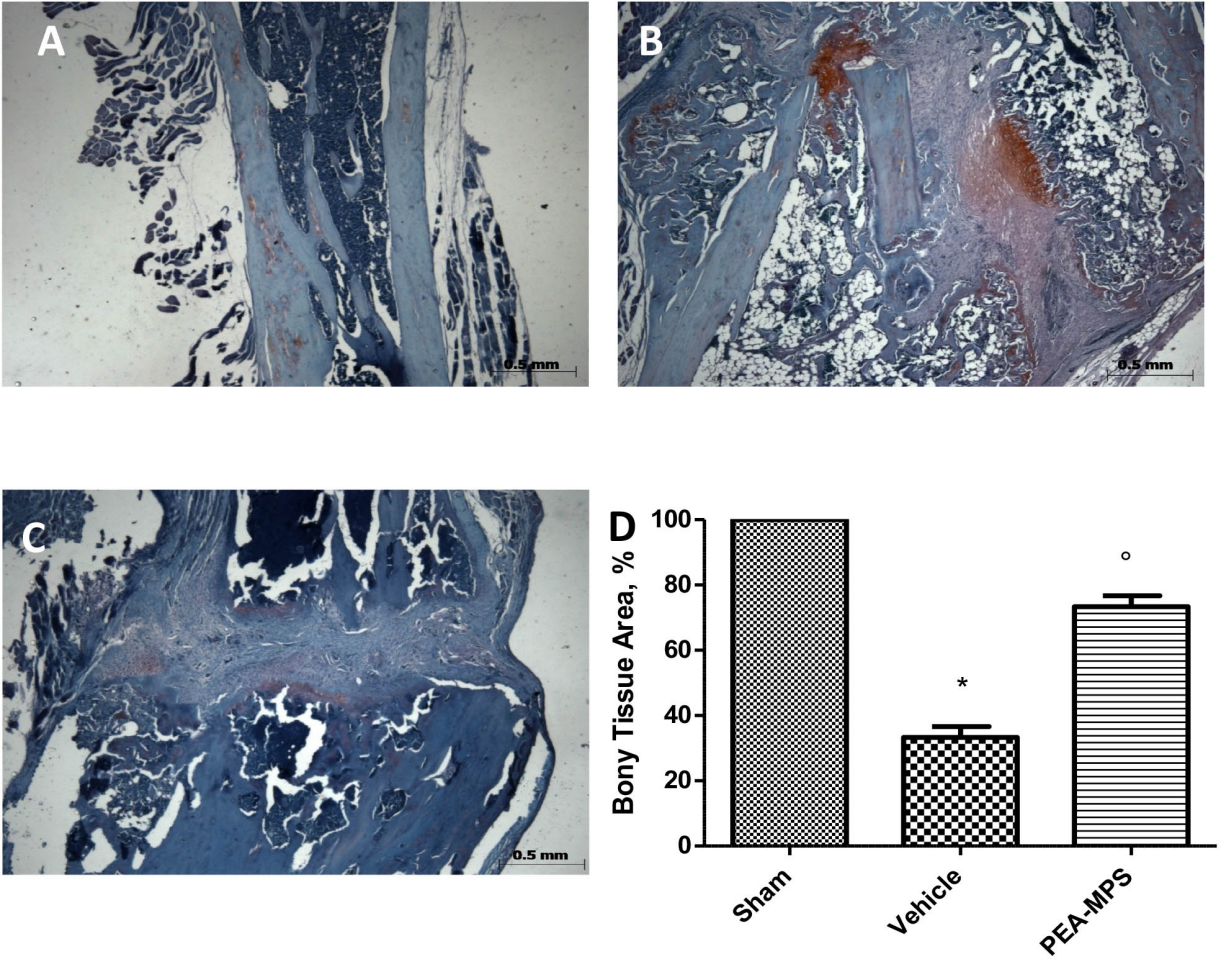
Efficacy of PEA-MPS on osteoclasts on tissue sections. Safranin O/fast green staining was used to evaluate skeletal cell differentiation and callus formation. PEA-MPS treatment accelerated osteogenesis and bone formation in the callus (C and D, and Figure c in S4 File) compared to the vehicle-treated mice (B and D, and Figure b in S4 File). A p-value less than 0.05 was considered significant. *p<0.05 vs. sham, ° *p*<0.05 vs vehicle.

Safranin O/fast green staining was utilized to evaluate the impact of PEA-MPS treatment on skeletal cell differentiation 28 days after fracture. Vehicle-treated mice displayed greater endochondral ossification (Fig 6B and 6D) compared to sham-treated mice (Fig 6A and 6D). PEA-MPS reduced the presence of osteoblasts in the fracture lesion (Fig 6C and 6D).

### Effect of PEA-MPS treatment on TNF-α and IL-1β expression

**Fig 7:**
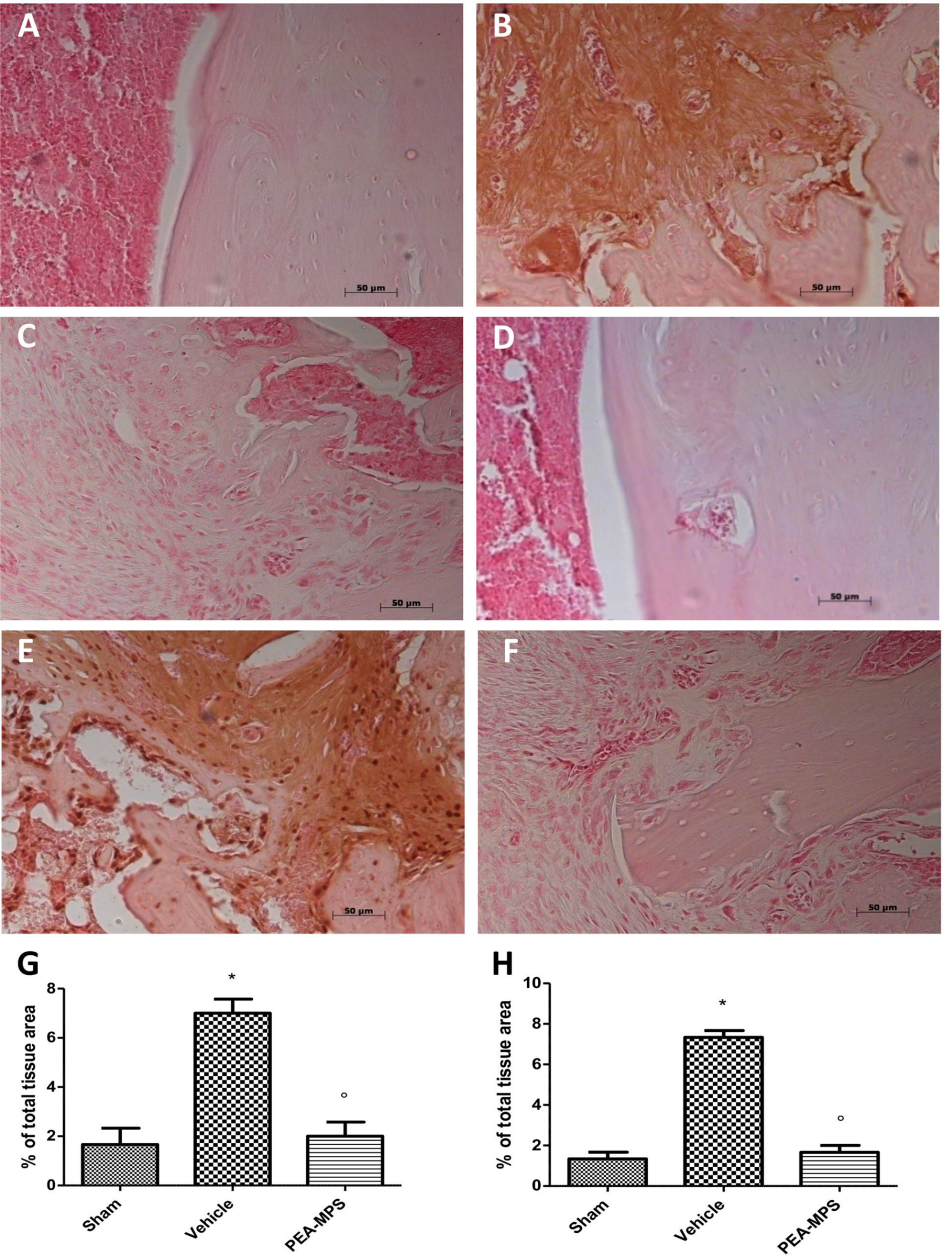
Efficacy of PEA-MPS on TNF-α and IL-1β expression. Twenty-eight days after surgery tissue taken from vehicle-treated mice displayed a substantial increase in TNF-α immunostaining (B and G, and Figure b in S5 File), compared to sham-treated mice(A and G, and Figure a in S5 File). At this time also IL-1β staining was increased (E and H, and Figure e in S5 File) with respect to sham-treated mice (D and H, and Figure d in S5 File). PEA-MPS treatment reduced staining for both markers (C, F and G, H, and Figure c and f in S5 File). A p-value less than 0.05 was considered significant. *p<0.05 vs. sham, ° *p*<0.05 vs vehicle.

At 28 days post-surgery tissues from vehicle-treated mice exhibited a substantial increase in TNF-α immunostaining (Fig 7B and 7G) and PEA-MPS treatment reduced this staining (Fig 7C and 7G). Moreover, mice subjected to distal tibia facture had increased immunoreactivity for IL-1β (Fig 7E and 7H) which was reduced by PEA-MPS treatment (Fig 7F and 7H). Sham-treated mice were immunonegative for both markers (Fig 7A, 7D, 7G and 7H).

### Effect of PEA-MPS treatment on nitrotyrosine and PAR formation

**Fig 8:**
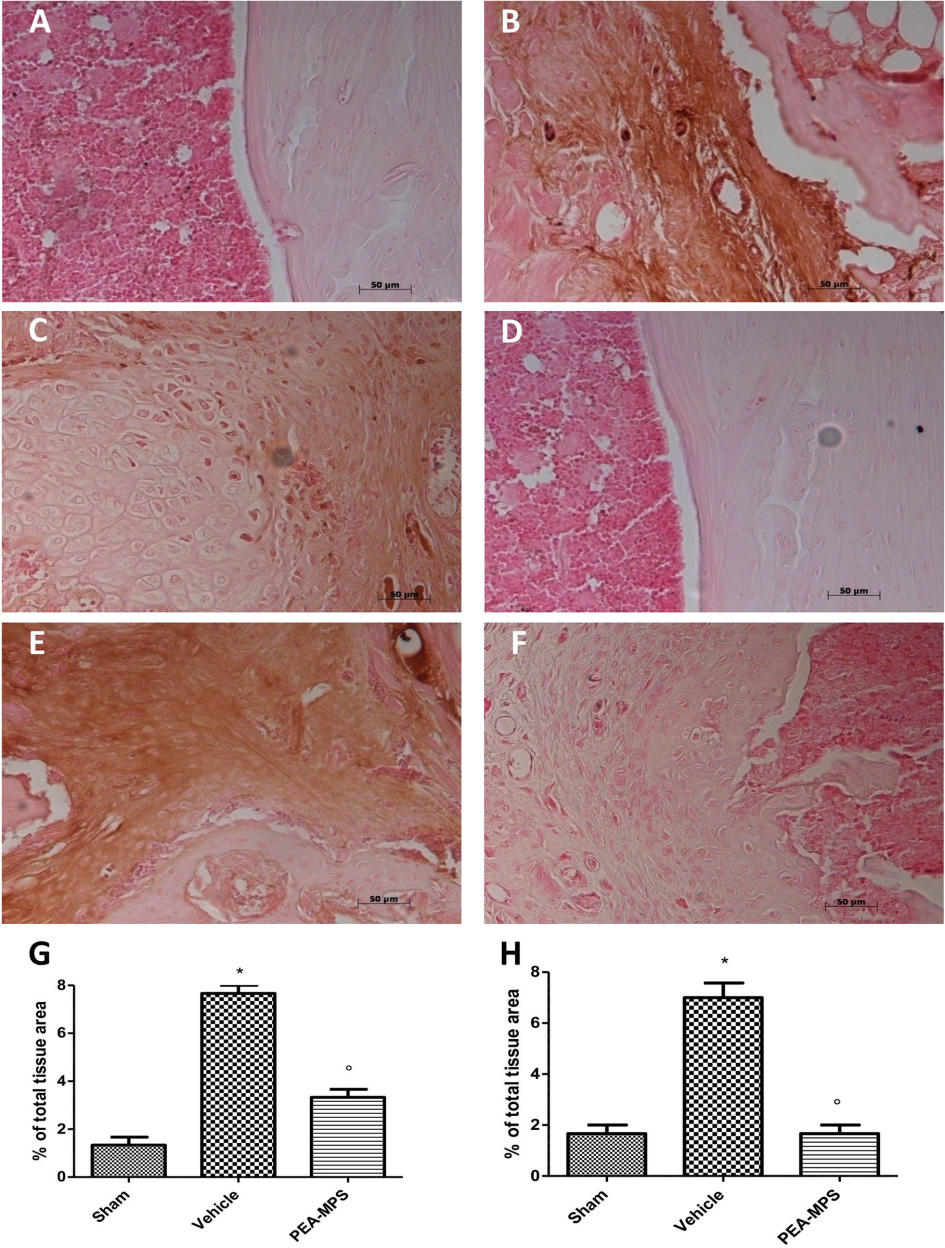
Efficacy of PEA-MPS on nitrotyrosine and PAR formation. Samples from vehicle-treated mice displayed positive nitrotyrosine staining (B and G, and Figure b in S6 File) and PEA-MPS treatment significantly reduced this effect (C and G, and Figure c in S6 File). Nuclear PAR staining was notably increased in inflammatory cells of vehicle mice (E and H, and Figure e in S6 File), which was reduced by PEA-MPS treatment (F and H, and Figure f in S6 File). Sham-treated mice failed to show positive staining (A, D for nitrotyrosine and G, H for PAR, respectively, Figure a and d in S6 File). A p-value less than 0.05 was considered significant. *p<0.05 vs. sham, ° *p*<0.05 vs vehicle.

The presence of nitrogen derivatives in animals at 28 days post-distal tibia fracture was assessed immunohistochemically as nitrotyrosine, a specific marker of nitrosative stress. Tissue from vehicle-treated mice exhibited positive nitrotyrosine staining (Fig 8B and 8G), which was significantly decreased by PEA-MPS treatment (Fig 8C and 8G). To examine poly(ADP-ribose)polymerase (PARP) activation, PAR development was examined immunohistochemically. PAR staining was significantly increased in the nuclei of inflammatory cells collected from vehicle-treated mice (Fig 8E and 8H), and PEA-MPS treatment markedly reduced this staining (Fig 8F and 8H). Sham-treated mice did not show any positive staining (Fig 8A, 8D, 8G and 8H).

### Effects of PEA-MPS treatment on Bax and Bcl-2 expression

**Fig 9:**
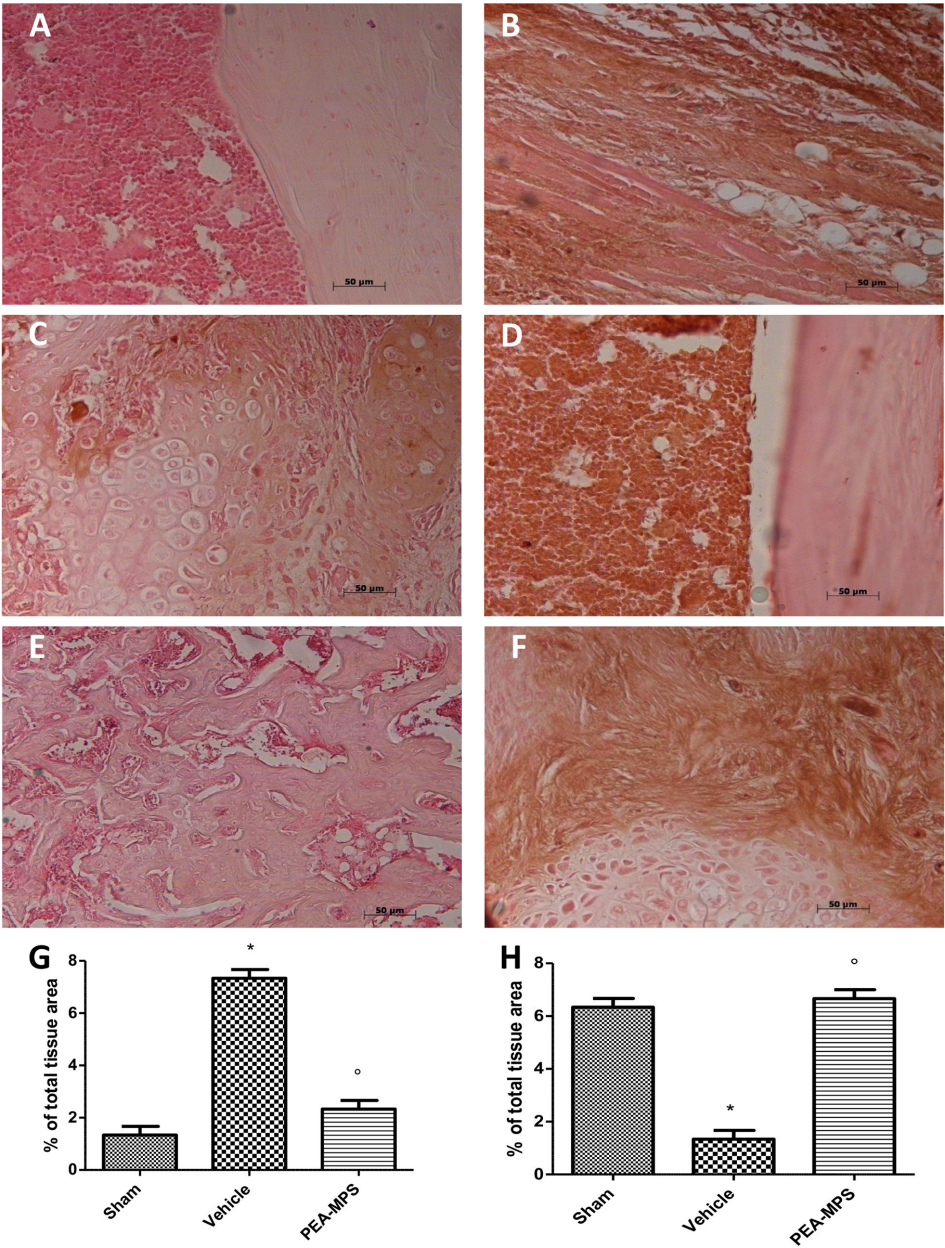
Efficacy of PEA-MPS on Bax and Bcl-2 expression. Twenty-eight days after surgery, tissues from vehicle-treated mice showed increased staining for Bax (B and G, and Figure b in S7 File) compared to sham mice (A and G, and Figure a in S7 File). PEA-MPS treatment decreased this staining (C and G, and Figure c in S7 File). Sham-treated mice exhibited positive Bcl-2 staining (D and H, and Figure d in S7 File) compared to vehicle-treated animals (E and H, and Figure e in S7 File). Moreover, PEA-MPS treatment reverted the inhibitory action of distal tibia fracture on Bcl-2 protein expression (F and H, and Figure f in S7 File). A p-value less than 0.05 was considered significant. *p<0.05 vs. sham, ° *p*<0.05 vs vehicle.

In order to test whether PEA-MPS treatment was able to modulate tibia distal fracture-induced apoptosis, we examined the expression of both the pro-apoptotic Bax and the anti-apoptotic Bcl-2 proteins. Vehicle-treated mice showed increased Bax staining (Fig 9B and 9G) compared to sham-treated mice (Fig 9A and 9G). PEA-MPS treatment decreased this staining (Fig 9C and 9G). In addition, sham-treated mice exhibited positive Bcl-2 staining (Fig 9D and 9H) compared to vehicle-treated animals (Fig 9E and 9H). Furthermore, PEA-MPS treatment reverted the inhibitory action of distal tibia fracture on Bcl-2 expression (Fig 9F and 9H).

## Discussion

The present study explored the effect of PEA-MPS treatment on CRPS-I in mice. The development of animal models displaying clinical signs substantially comparable to human CRPS-I with the same evolution as frequently seen in the clinic has been an area of active examination. Distal tibia fracture is the most widely used mouse model of CRPS-I-associated chronic pain due to its ability to increase local release of cytokines and pro-inflammatory neuropeptides [31]. Such bone fracture may be the event that triggers and maintains the initial phases of the pathology, causing allodynia and hyperalgesia [22]. PEA-MPS treatment reduced the mechanical hyperalgesia and thermal nociception. Our results revealed also an action of oral PEA-MPS in the process of fracture healing. These animals showed a decreased inflammatory response during the early phase and an increase in the degree of fibrosis, cartilaginous callus formation and woven bone remodelling during the late phase. In the primary stage of the inflammatory process, mast cell accumulation in injured tissues plays a central role [32]. In the skin of the affected extremity CRPS-I patients show increased tryptase, suggesting an increase in mast cell accumulation and degranulation [33]. Because mast cells release a number of inflammatory mediators such as NGF [34] in injured tissues, we hypothesized that mast cell degranulation is involved in the nociceptive sensitization in this tibia fracture model of CRPS-I. PEA-MPS oral treatment reduced mast cells and, in parallel, significantly limited NGF expression in the inflamed tissue.

MMPs have important roles in bone repair and development, and participate in the interaction between skeletal progenitors and inflammatory cells [29, 35]. MMP9 participates also during the inflammatory phases of repair [36]. PEA-MPS oral administration significantly up-regulated MMP9 expression compared to vehicle-treated mice. MMP9 is expressed both by osteoclasts and bone marrow-derived myeloid cells, which are involved in extracellular matrix remodelling during bone repair [37]. PEA-MPS treatment clearly accelerated endochondral ossification, whereas numerous uncalcified chondrocytes–implicated in delayed healing—persisted at the fracture site in untreated mice.

In the first phase of fracture repair, primary haemorrhage within fracture sites progresses into a hematoma, with infiltrating inflammatory cells (including macrophages) [38]. This infiltrate induces the activation of inflammatory cascades through the secretion of numerous cytokines. Macrophages produce pro-inflammatory cytokines such as TNF-α and IL-1 [39]. PEA-MPS treatment reduced up-regulation of these two cytokines. In the cascade of events which accompany the later phases of CRPS-I, metabolic alterations due to a disturbed capillary exchange exacerbate and maintain the clinical features of CRPS-I. In this setting, up-regulation of oxygen free radicals such as superoxide anion and peroxynitrite support the inflammatory process [40]. These radicals cause oxidization of sulfhydryl groups, lipid peroxidation and nitration of tyrosine residues. Lipid peroxidation and nitrotyrosine expression were elevated in our distal tibia fracture mice, and treatment with PEA-MPS significantly reduced nitrotyrosine immunostaining. Peroxynitrite can also activate the DNA repair enzyme PARP, which synthesizes chains of ADP-ribose in reply to single-strand DNA breaks. NAD+ is required for this reaction, and hyper-activation of PARP can reduce cellular reserves of NAD+ and lead to ATP depletion, ultimately resulting in cellular dysfunction and death. In the present study, PEA-MPS treatment decreased PARP activity. Finally, tissue apoptosis may serve as a marker for CRPS-I [41, 42]. Our data show that PEA-MPS treatment reduced tibia fracture-induced Bax expression and increased Bcl-2 expression that was reduced in tibia fracture mice.

## Conclusions

Collectively, the findings described here demonstrate that 28 days after tibia fracture induction PEA-MPS treatment, by attenuating the inflammatory response, allodynia and hyperalgesia, may be an innovative pharmacological approach for treatment of CRPS-I.

## Supporting information

S1 File **a** Original hematoxylin/eosin image for Sham group **b** Original hematoxylin/eosin image for Fracture group **c.** Original hematoxylin/eosin image for PEA-MPS group **d** Original Masson trichrome image for Sham group **e** Original Masson trichrome image for Fracture group **f** Original Masson trichrome image for PEA-MPS group.
(PDF)

S2 File **a** Original mast cell image for Sham group **b** Original mast cell image for Fracture group **c.** Original mast cell image for PEA-MPS group **d** Original immunohistochemical image for NGF for Sham group **e** Original immunohistochemical image for NGF for Fracture group **f** Original immunohistochemical image for NGF for PEA-MPS group.
(PDF)

S3 File **a** Original immunohistochemical image for MMP9 for Sham group **b** Original immunohistochemical image for MMP9 for Fracture group **c.** Original immunohistochemical image for MMP9 for PEA-MPS group **d** Original cartilage image for Sham group **e** Original cartilage image for Fracture group **f** Original cartilage image for PEA-MPS group.
(PDF)

S4 File **a** Original safranin O/fast green image for osteoclast for Sham group **b** Original safranin O/fast green image for osteoclast for Fracture group **c.** Original safranin O/fast green image for osteoclast for PEA-MPS group.
(PDF)

S5 File **a** Original immunohistochemical image for TNF-α for Sham group **b** Original immunohistochemical image for TNF-α for Fracture group **c.** Original immunohistochemical image for TNF-α for PEA-MPS group **d** Original immunohistochemical image for IL-1β for Sham group **e** Original immunohistochemical image for IL-1β for Fracture group **f** Original immunohistochemical image for IL-1β for PEA-MPS group.
(PDF)

S6 File **a** Original immunohistochemical image for nitrotyrosine for Sham group **b** Original immunohistochemical image for nitrotyrosine for Fracture group **c.** Original immunohistochemical image for nitrotyrosine for PEA-MPS group **d** Original immunohistochemical image for PAR for Sham group **e** Original immunohistochemical image for PAR for Fracture group **f** Original immunohistochemical image for PAR for PEA-MPS group.
(PDF)

S7 File **a** Original immunohistochemical image for Bax for Sham group **b** Original immunohistochemical image for Bax for Fracture group **c.** Original immunohistochemical image for Bax for PEA-MPS group **d** Original immunohistochemical image for Bcl-2 for Sham group **e** Original immunohistochemical image for Bcl-2 for Fracture group **f** Original immunohistochemical image for Bcl-2 for PEA-MPS group.
(PDF)
