# Correction: Effect of a new formulation of micronized and ultramicronized N-palmitoylethanolamine in a tibia fracture mouse model of complex regional pain syndrome

**DOI:** 10.1371/journal.pone.0201501

**Published:** 2018-07-25

**Authors:** Roberta Fusco, Enrico Gugliandolo, Michela Campolo, Maurizio Evangelista, Rosanna Di Paola, Salvatore Cuzzocrea

The authors would like to correct the following mistakes which were inadvertently included in the publication [1].

In the “Experimental groups” paragraph of the Materials and Methods section, the dose of PEA-MPS administrated to the animals was 10 mg/Kg, and it was chosen based on previous experiments as indicated ([2]; reference 23 of the original article). The sentence “300 mg/kg PEAm and 600 mg/kg PEA-um®” incorrectly included into the manuscript was referring to the composition of the PEA-MPS (300 mg PEAm and 600 mg PEA-um®). Moreover, the calculation to know the dose of PEA-MPS that should be assumed by human, included into the manuscript, is incorrect. According to the formula applied ([3]; reference 24 of the original article), for the conversion from animal to human, the dose of PEA-MPS that should be taken would be about 48,6 mg for a 60 Kg human. Human Equivalent Dose PEA-MPS (mg/kg) = (10x[3/37]) = 0,81 mg/Kg, thus for a 60 Kg human, the dose is 48,6 mg.

The correct “Experimental groups” paragraph is as follows:

## Experimental groups

Mice were randomly divided into the following groups (n = 10 for each group):

- Sham + vehicle group: vehicle solution (carboxymethylcellulose 1.5% wt/vol in saline) was administered orally for 28 days.
- Sham + PEA-MPS group: mice were treated orally daily with 10 mg/Kg PEA-MPS (300 mg PEAm and 600 mg PEA-um®) for 28 days.
- Fracture + vehicle group: vehicle solution (carboxymethylcellulose 1.5% wt/vol in saline) was administered orally daily 1 h after surgery for 28 days.
- Fracture + PEA-MPS group: mice were treated orally daily with 10 mg/Kg PEA-MPS (300 mg PEAm and 600 mg PEA-um®) 1 h after surgery for 28 days.

The dose of PEA-MPS was chosen based on previous experiments ([2]; reference 23 in the original publication).

In the previous study ([2]; reference 23 in the original publication), we have demonstrated the beneficial effects of PEA in reducing edema formation and thermal hyperalgesia in carrageenan-induced inflammation in the rat paw. These results show the differential effects exerted on the degree of inflammation by micronized PEA-m and ultramicronized PEA-um, vs nonmicronized PeaPure.

We have done the calculation to know the dose of PEA-MPS that should be assumed by a human. According to the formula applied for the conversion from animal to human, the dose of PEA-MPS that should be taken would be about 48,6 mg for a 60 Kg human. Human Equivalent Dose PEA-MPS (mg/kg) = (10x[3/37]) = 0,81 mg/Kg, thus, for a 60 Kg human, the dose is 48,6 mg.

The minimum number of animals for each group was calculated using the statistical test a priori power analyzes of the G-power software. This statistical test provides an efficient method for determining the sample size necessary to perform the experiment before the experiment the same is actually conducted.

In addition, the primary data underlying quantitative results were not included with the published article. The primary data are included below in S1 File.

The authors would like to apologize for these mistakes.

## Supporting information

S1 File Raw dataset. (XLSX)
