# Supplementary figures and images for: Effect of a new formulation of micronized and ultramicronized N-palmitoylethanolamine in a tibia fracture mouse model of complex regional pain syndrome
Source: PLoS One. 2017 Jun 8;12(6):e0178553. doi: 10.1371/journal.pone.0178553 (PMC5464592; doi:10.1371/journal.pone.0178553)

Fig. S1a

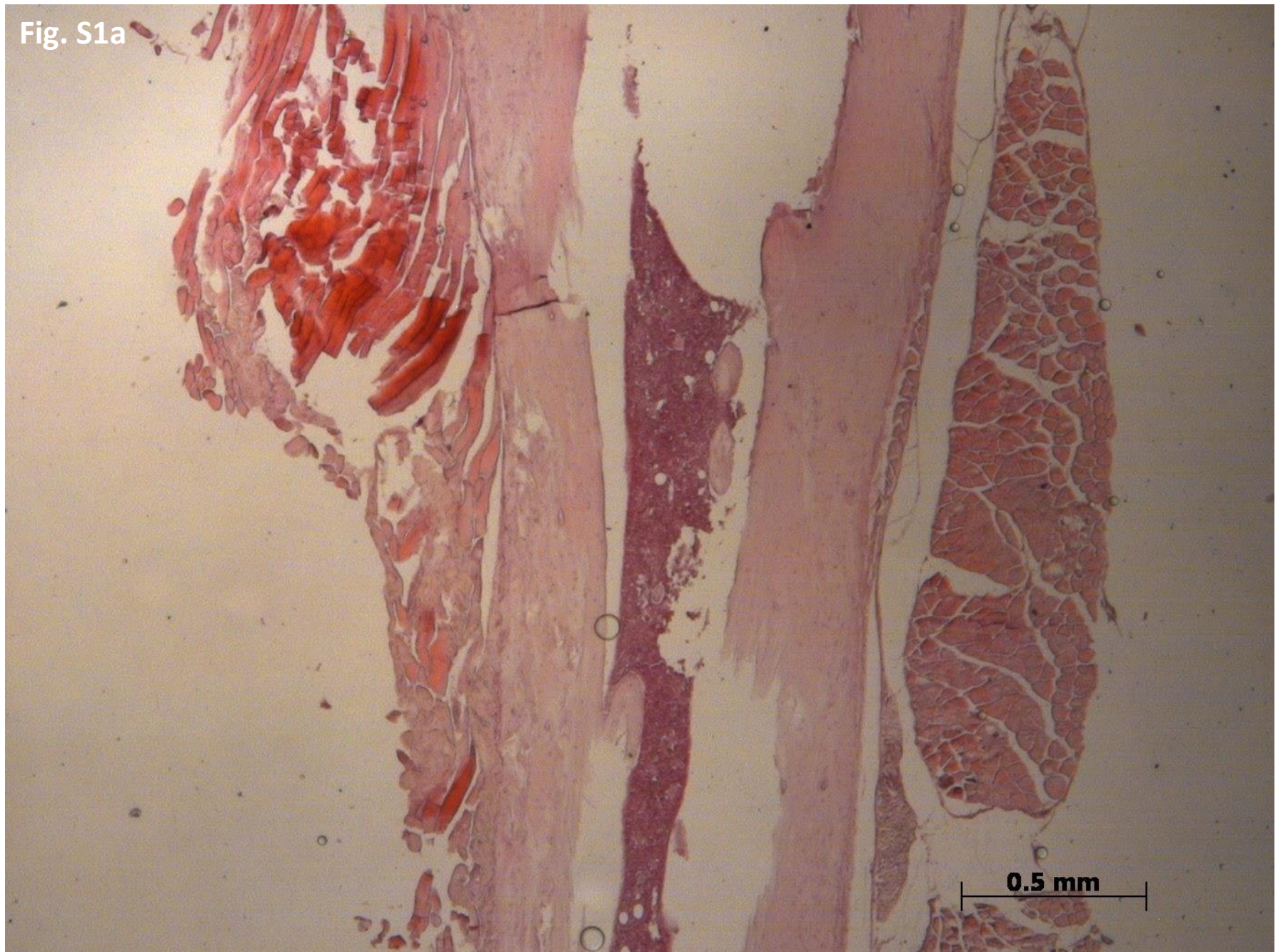

Fig. S1b

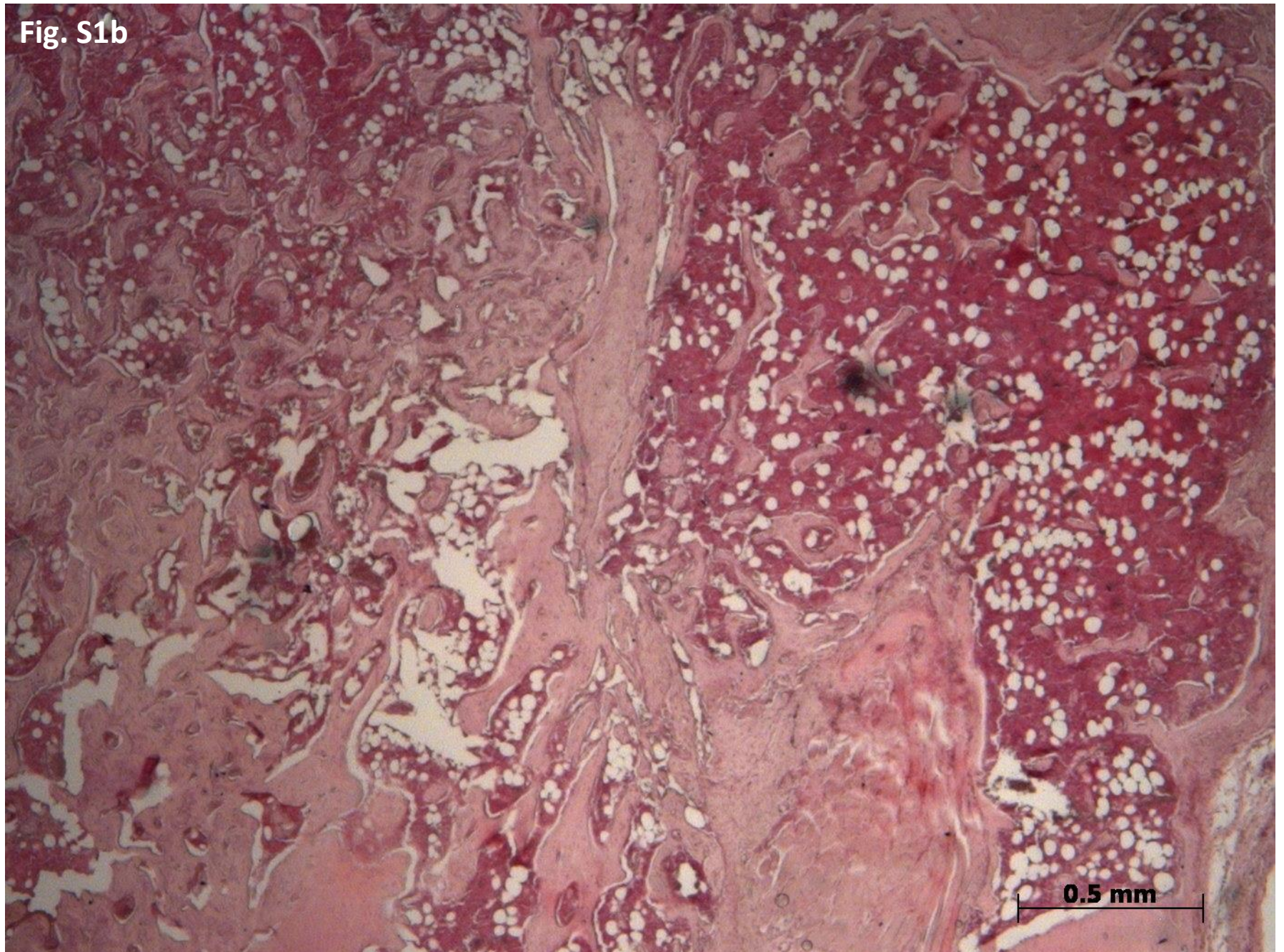

Fig. S1c

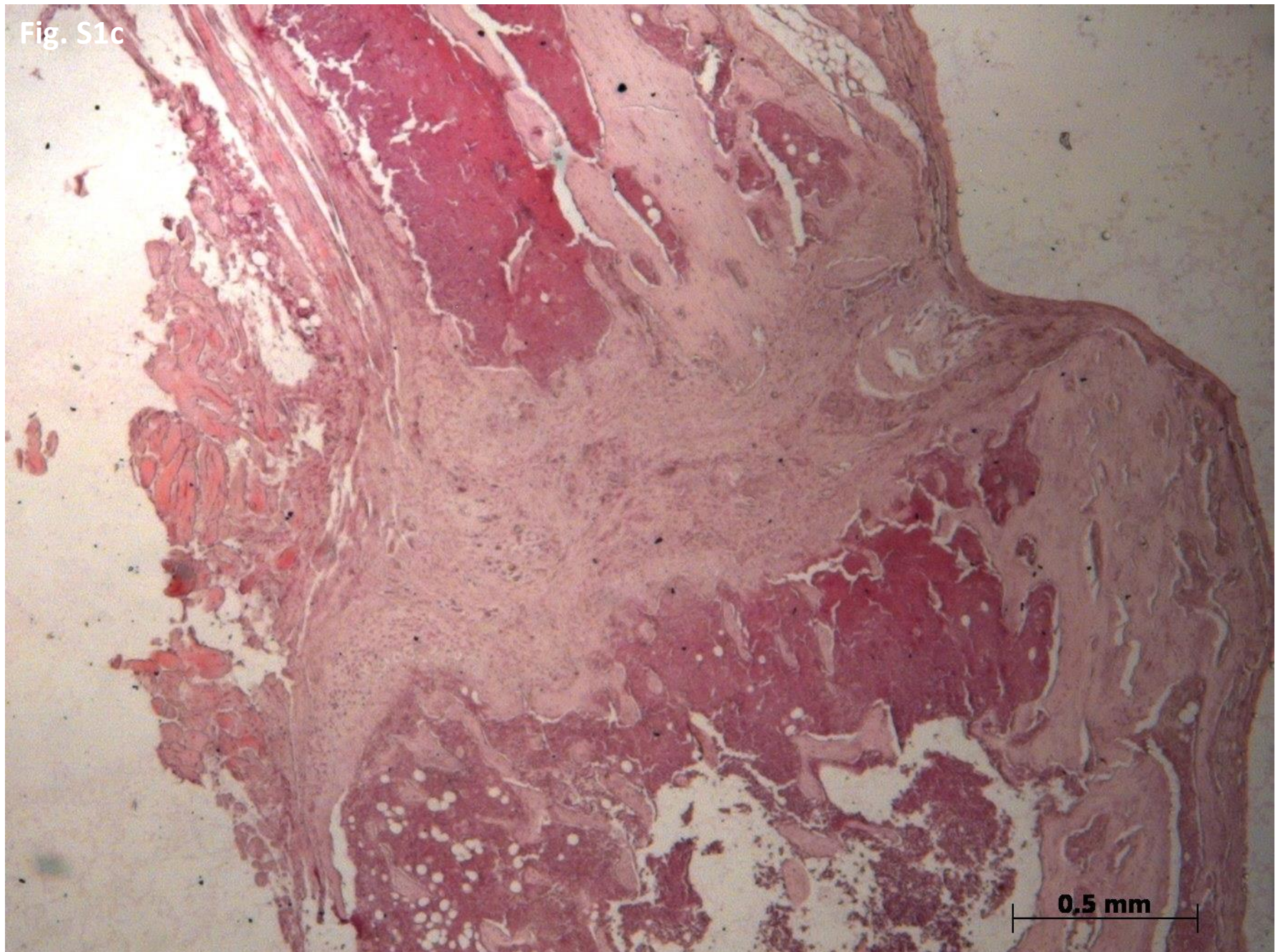

Fig. S1d

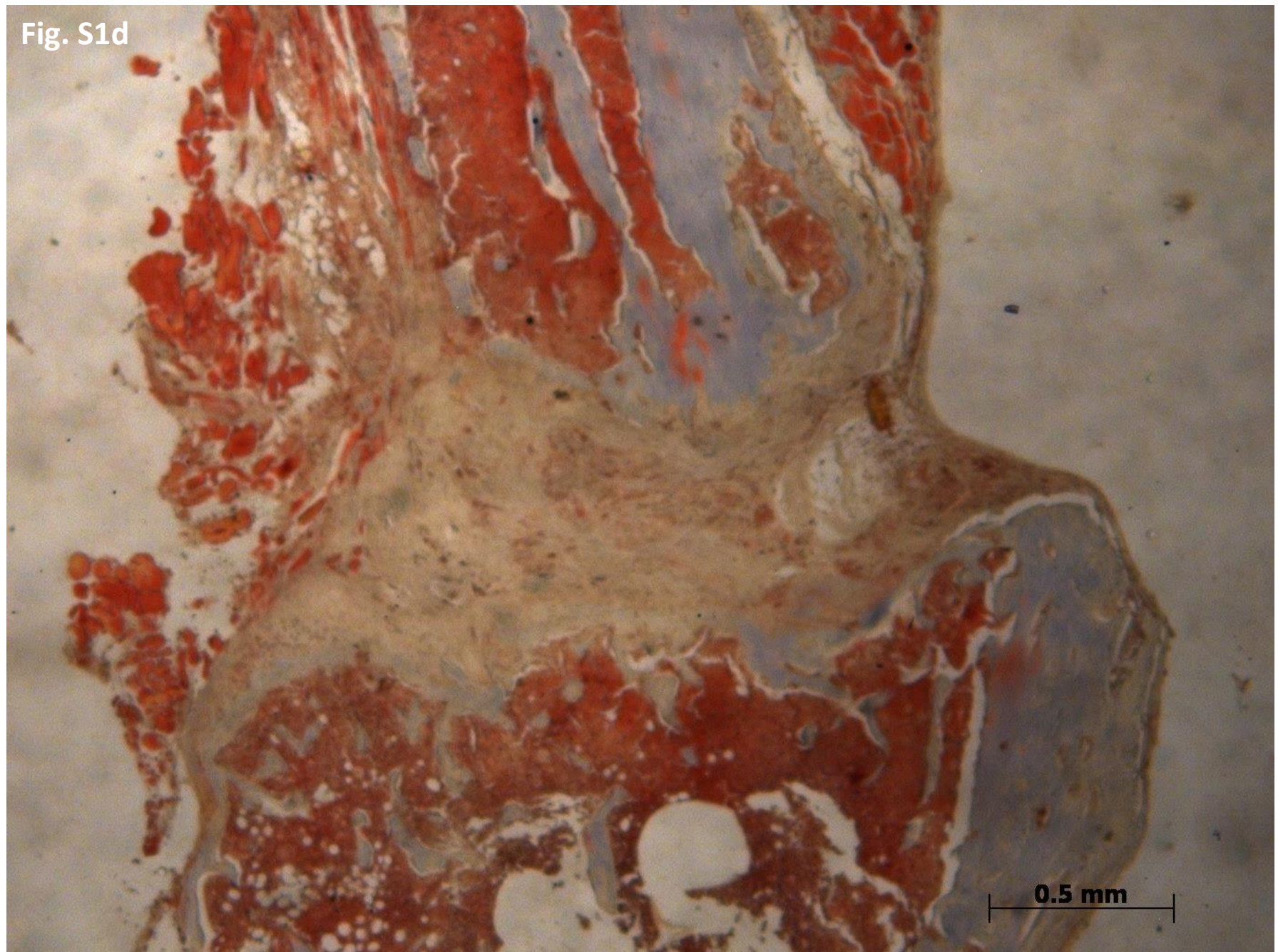

Fig. S1e

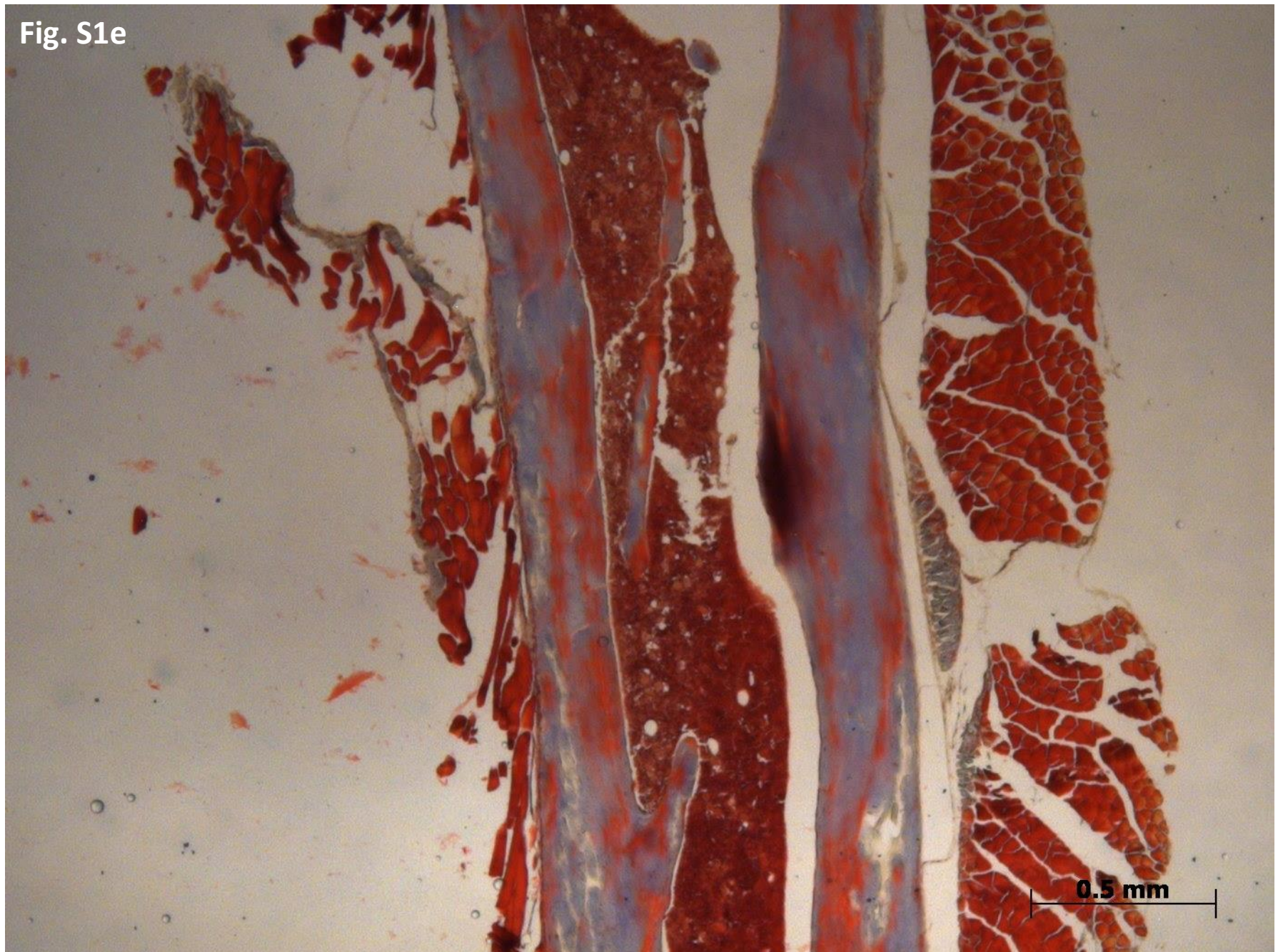

Fig. S1f

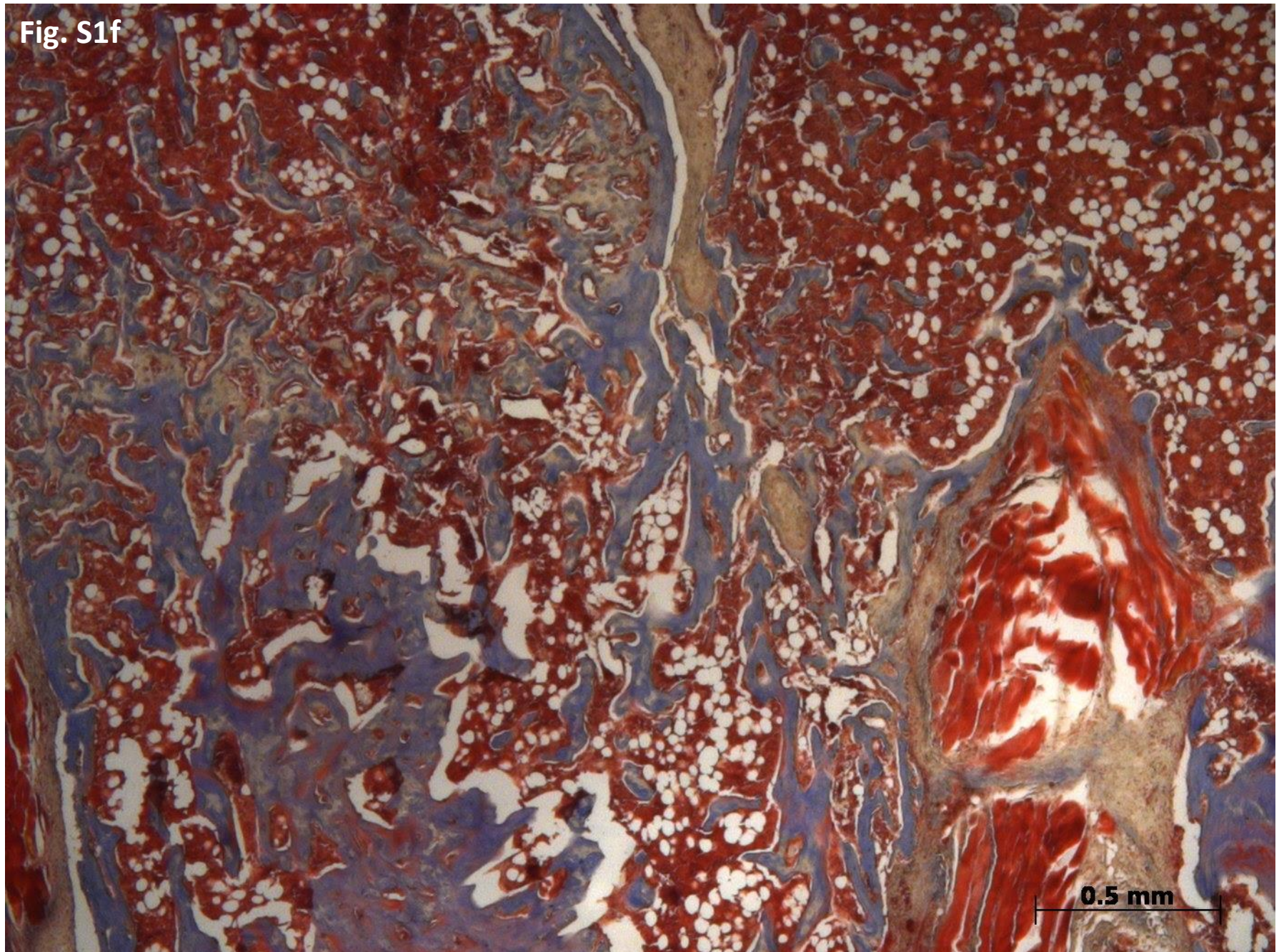

Supplement: S1 File — a Original hematoxylin/eosin image for Sham group b Original hematoxylin/eosin image for Fracture group c. Original hematoxylin/eosin image for PEA-MPS group d Original Masson trichrome image for Sham group e Original Masson trichrome image for Fracture group f Original Masson trichrome image for PEA-MPS group. (PDF) [file pone.0178553.s001.pdf]

Fig. 2Sa

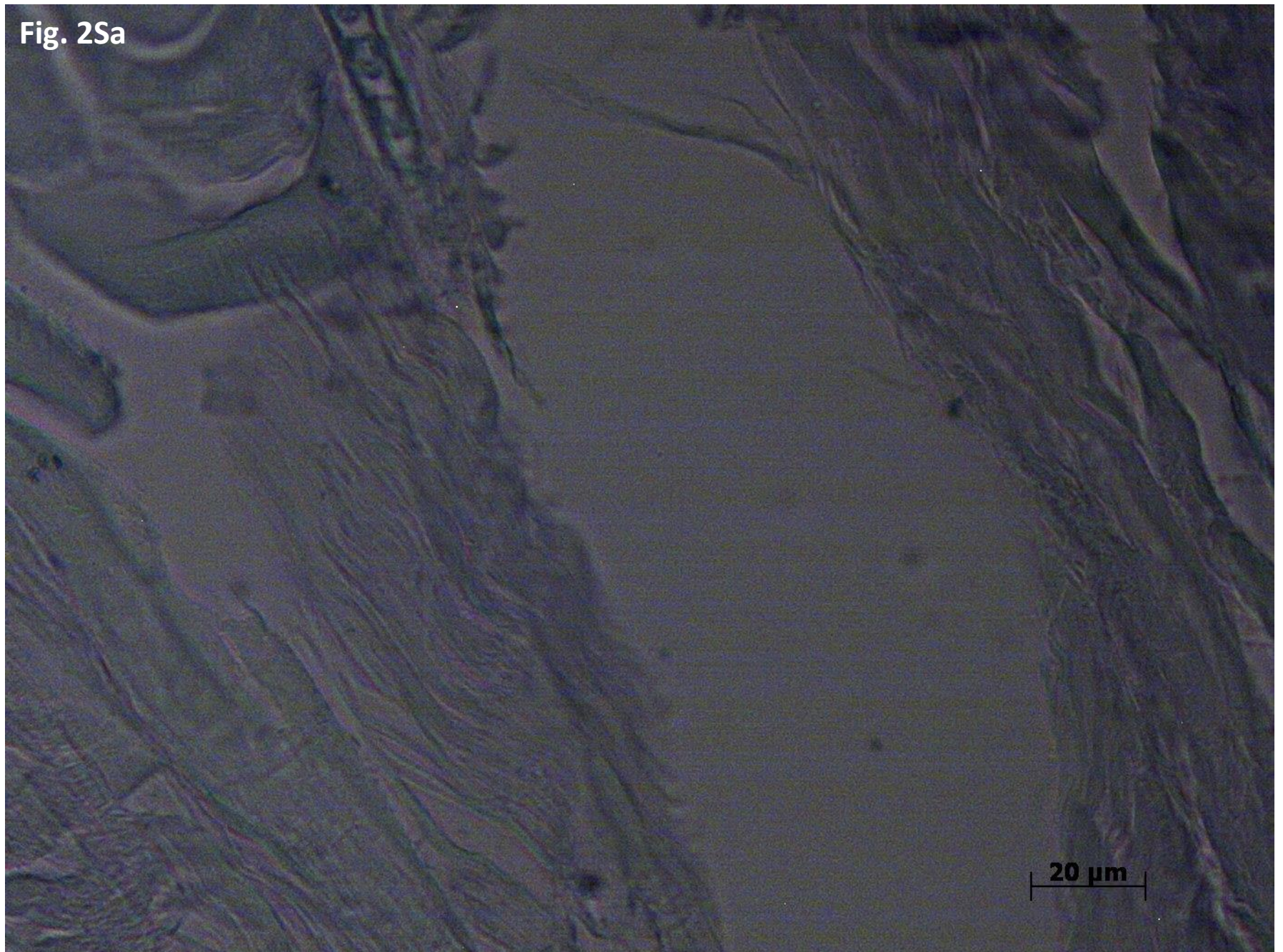

Fig. 2Sb

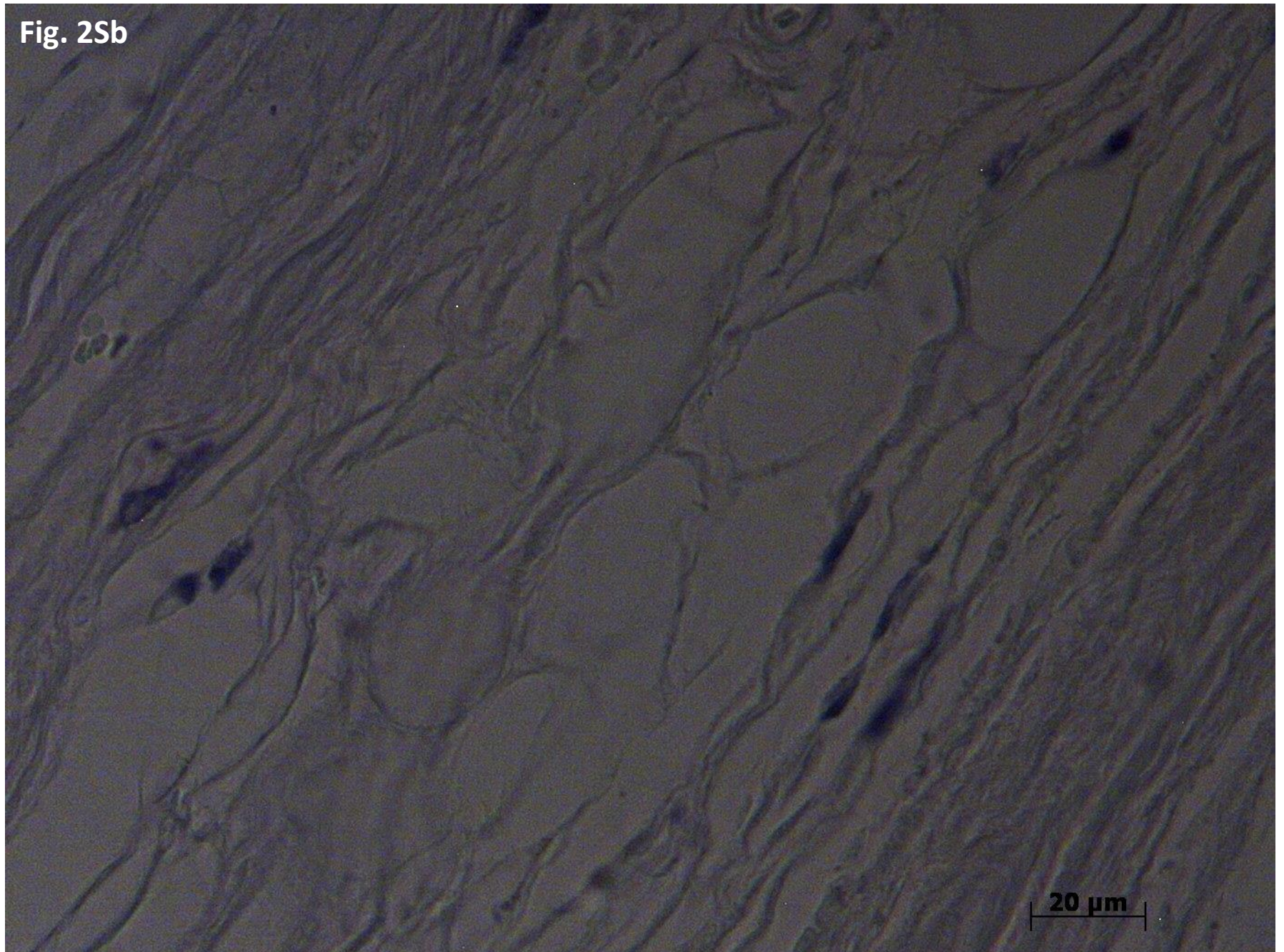

Fig. 2Sc

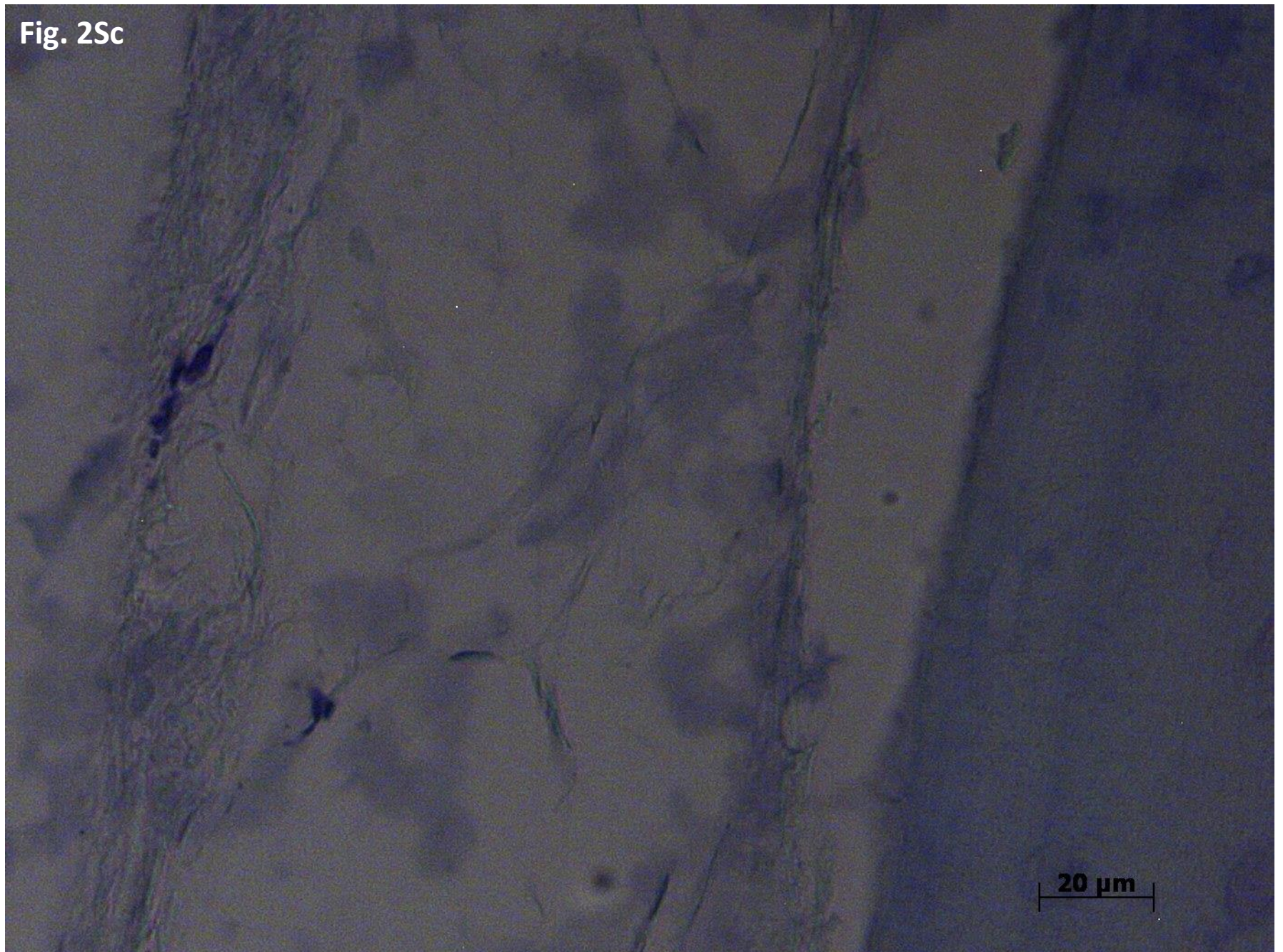

Fig. 2Sd

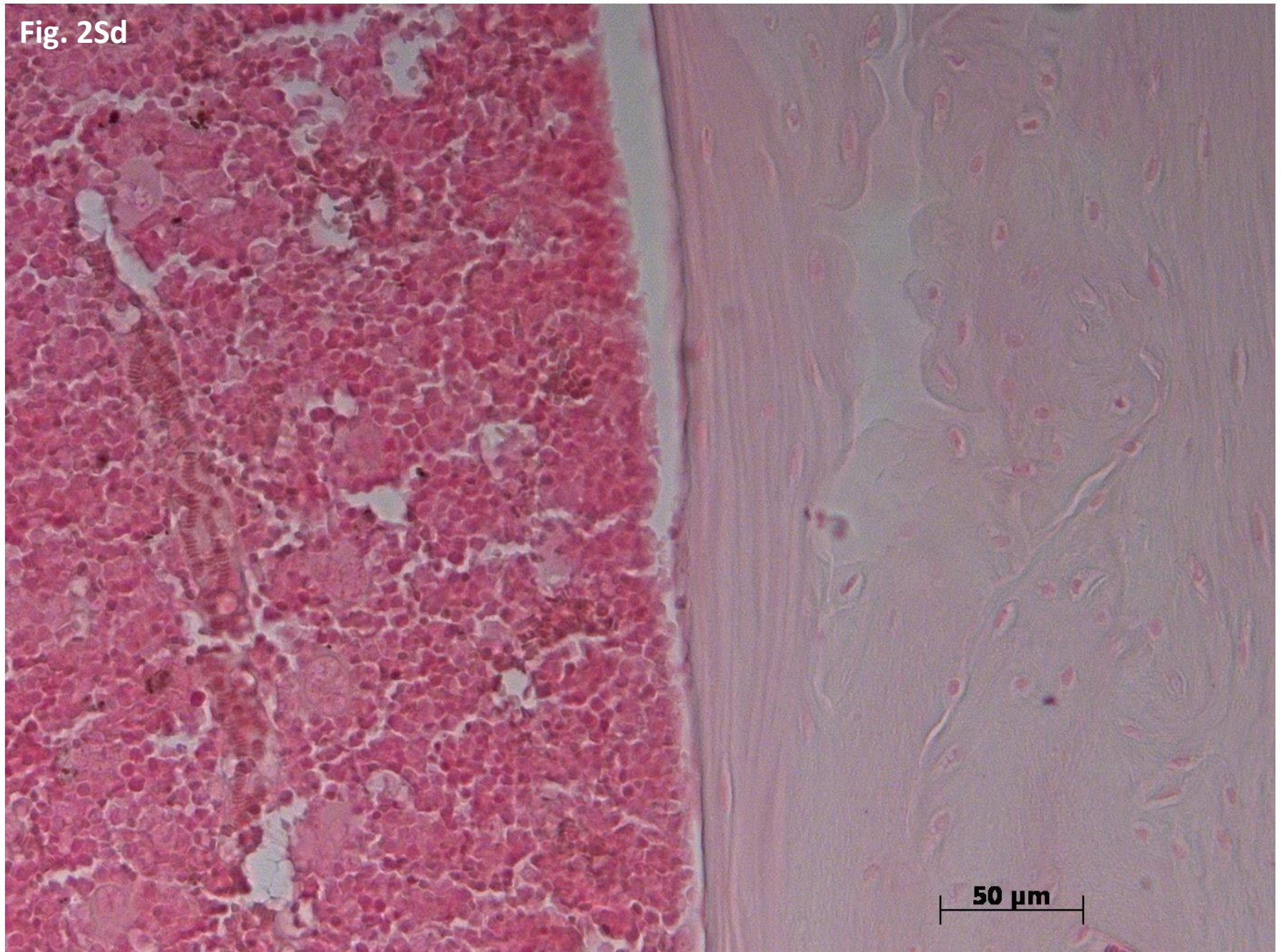

Fig. 2Se

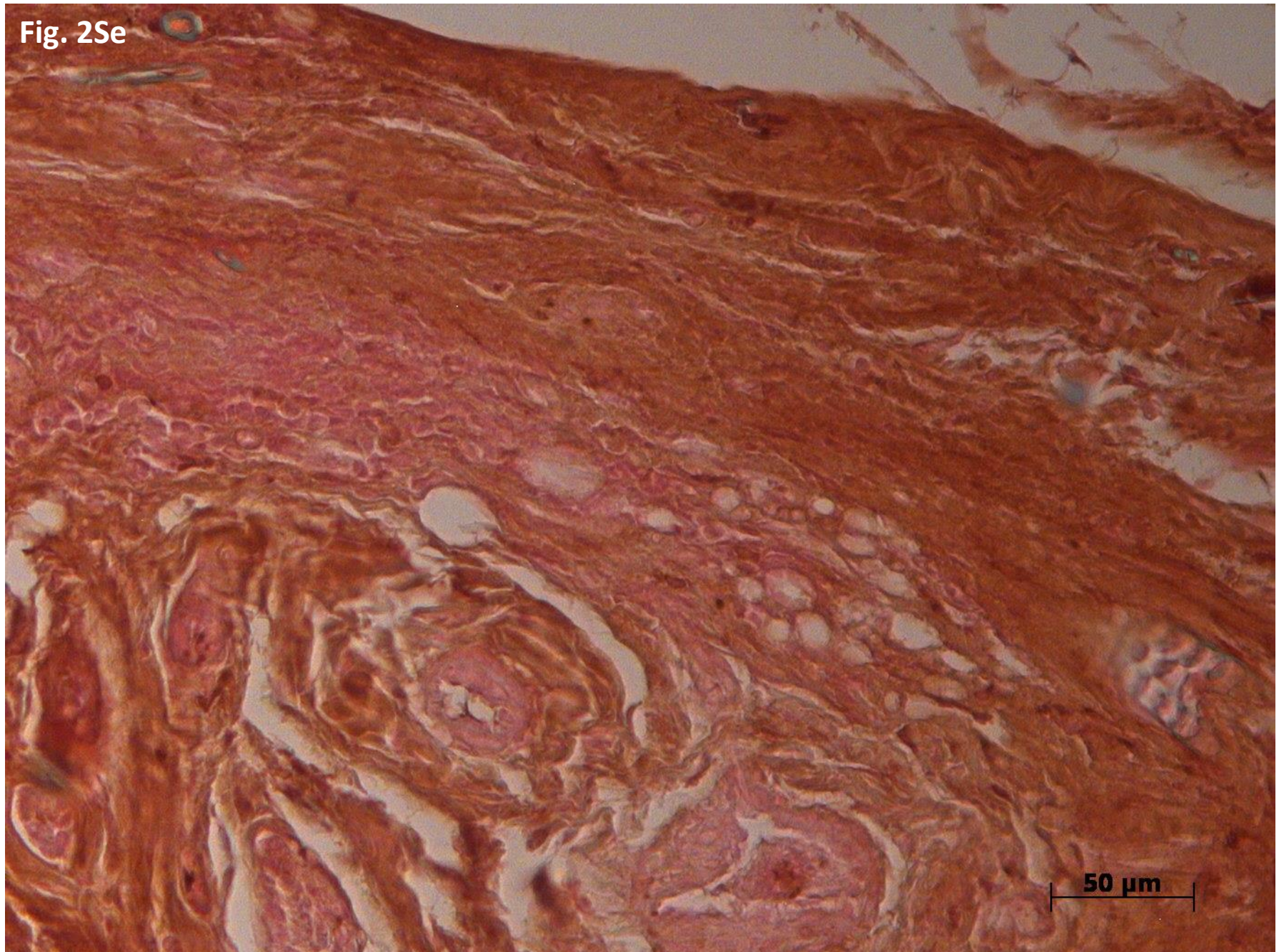

Fig. 2Sf

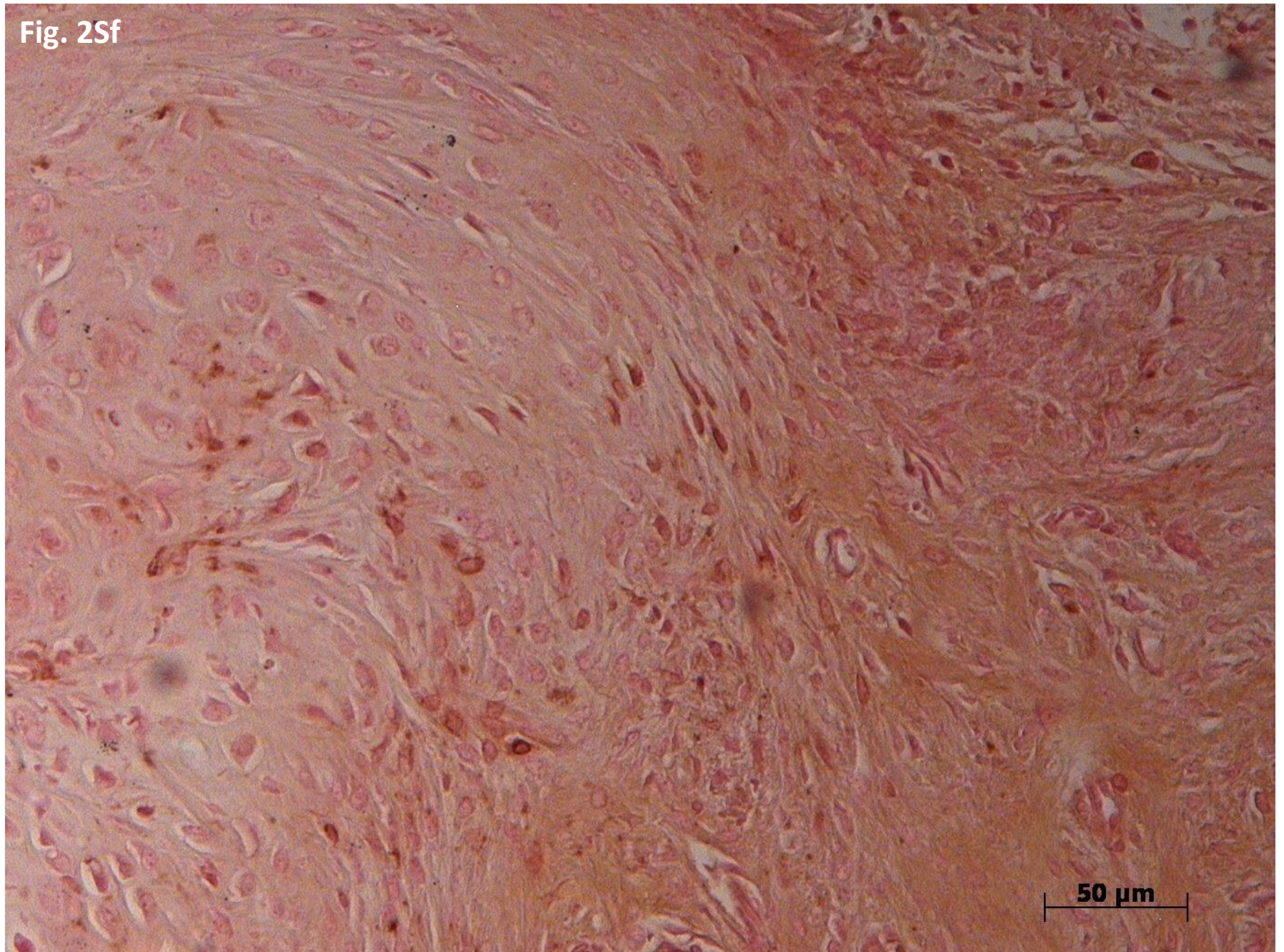

Supplement: S2 File — a Original mast cell image for Sham group b Original mast cell image for Fracture group c. Original mast cell image for PEA-MPS group d Original immunohistochemical image for NGF for Sham group e Original immunohistochemical image for NGF for Fracture group f Original immunohistochemical image for NGF for PEA-MPS group. (PDF) [file pone.0178553.s002.pdf]

Fig. 3a

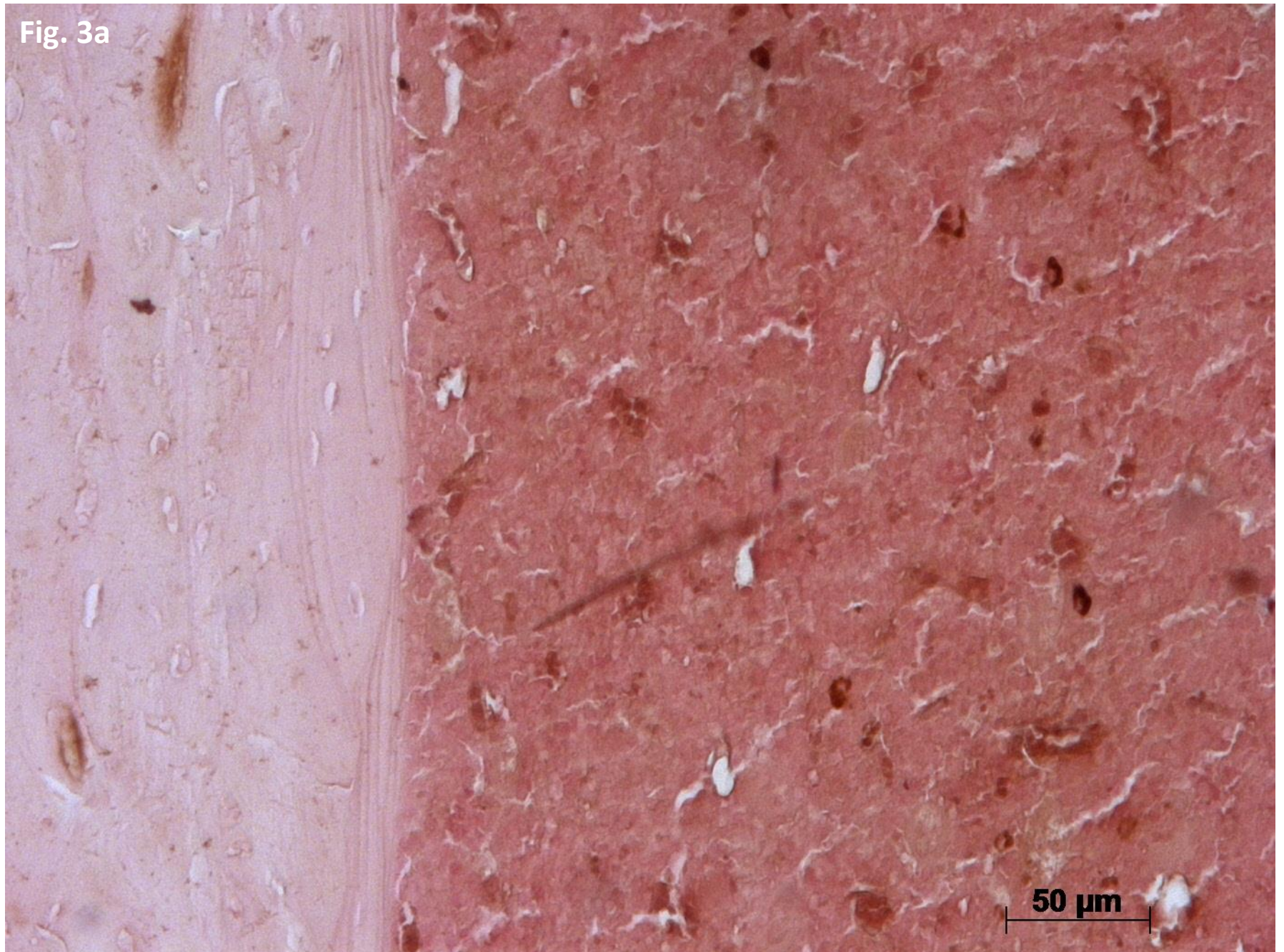

**Fig. 3b**

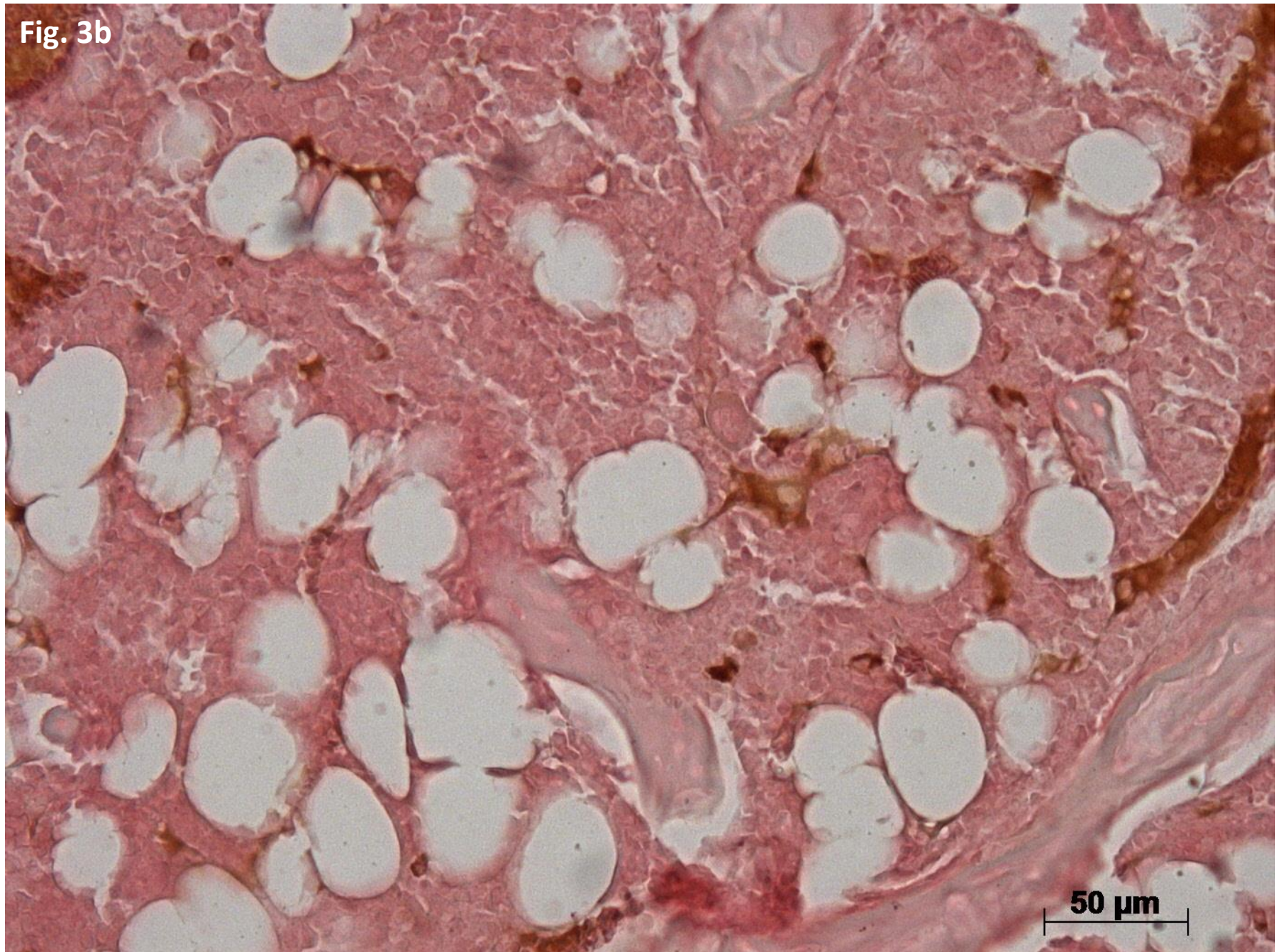

Fig. 3c

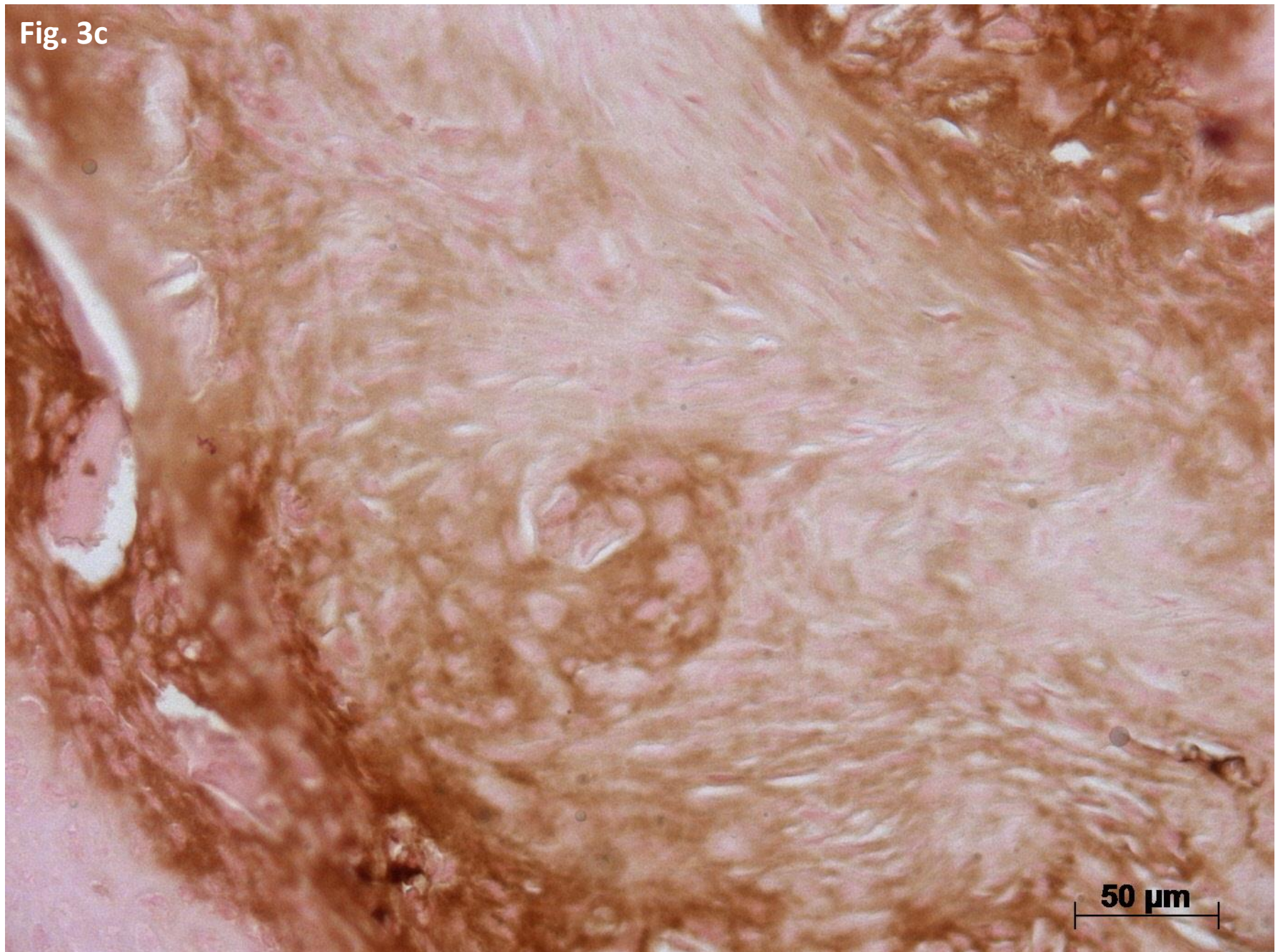

Fig. 3d

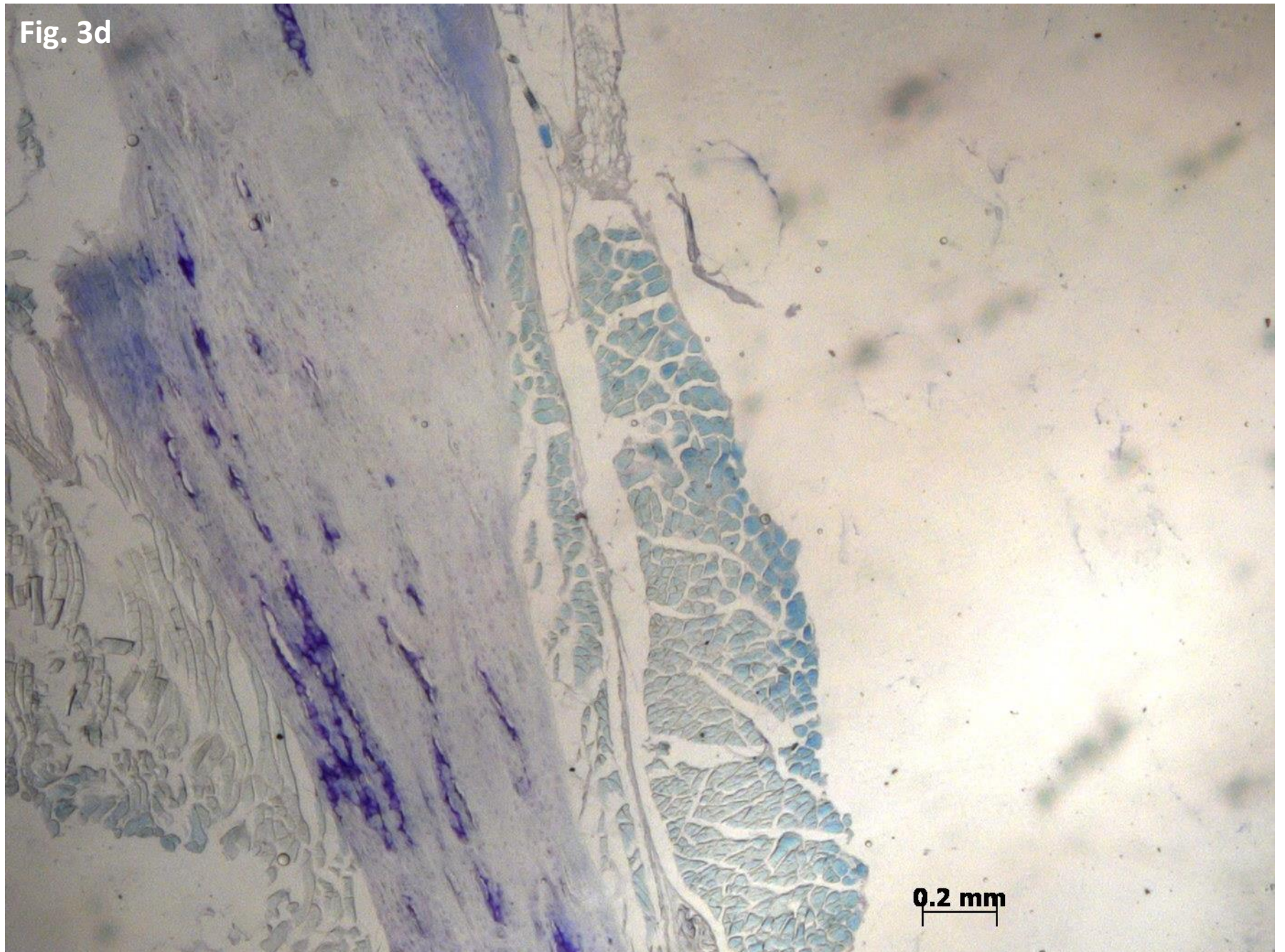

Fig. 3e

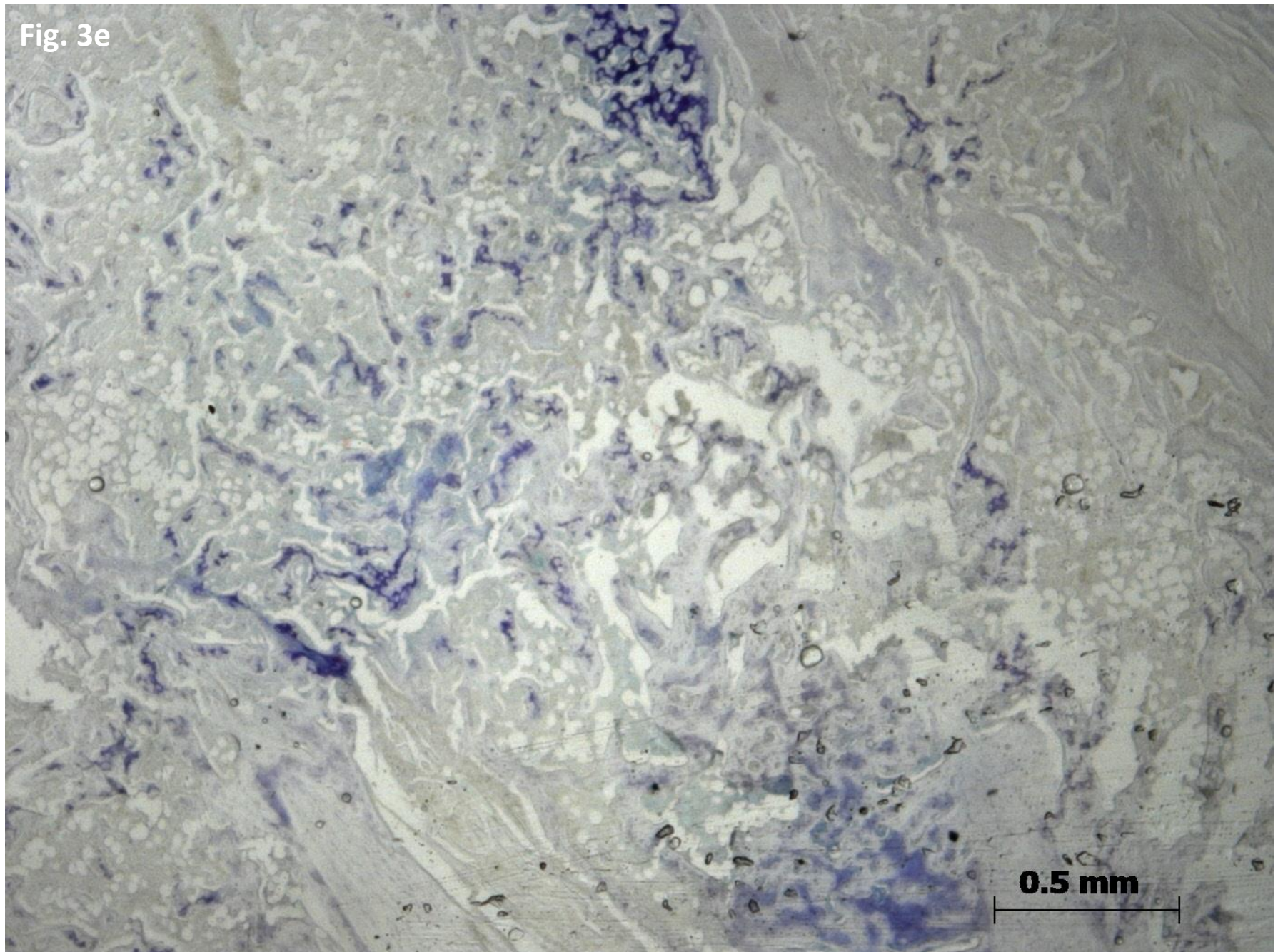

Fig. 3f

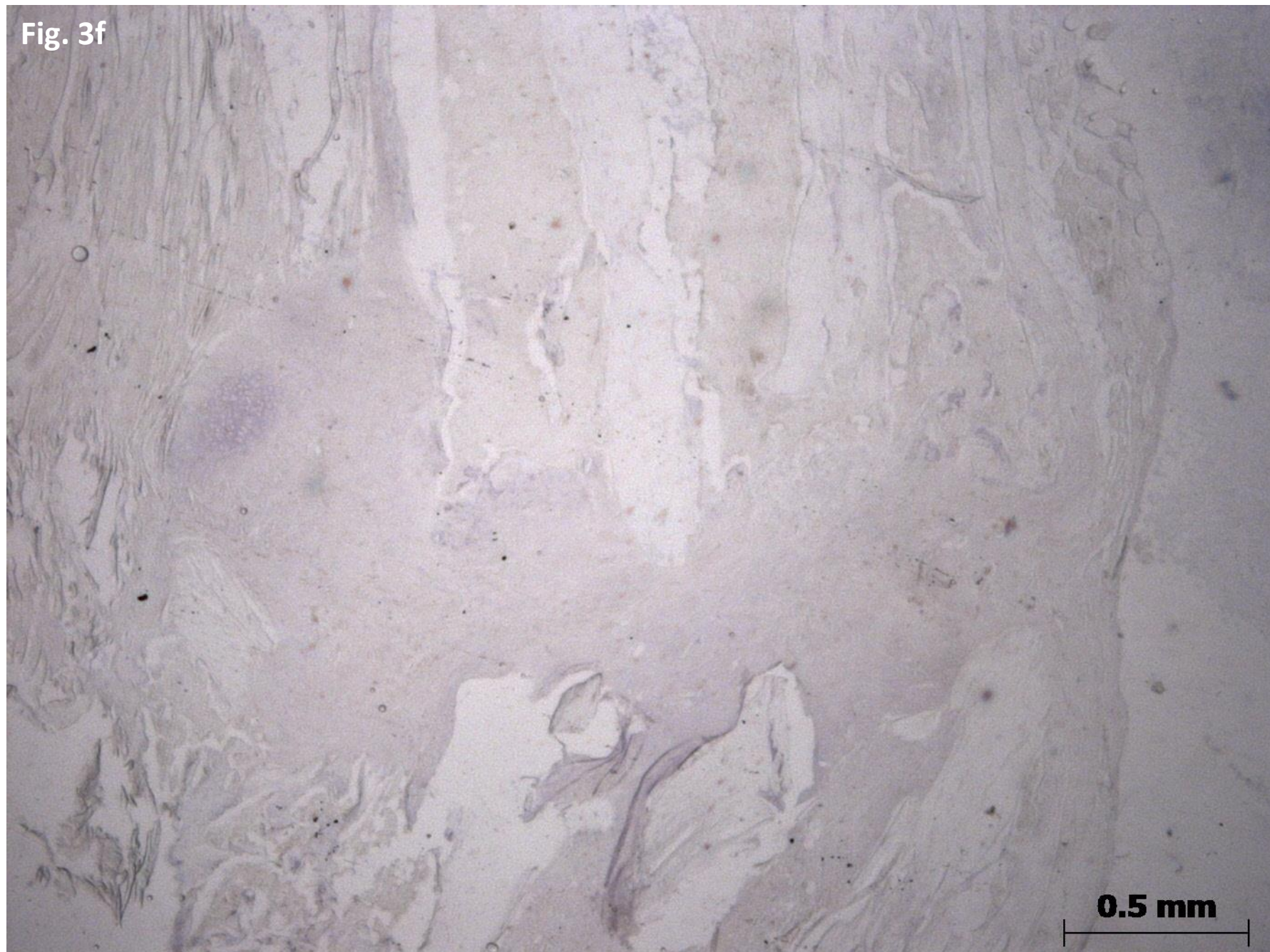

Supplement: S3 File — a Original immunohistochemical image for MMP9 for Sham group b Original immunohistochemical image for MMP9 for Fracture group c. Original immunohistochemical image for MMP9 for PEA-MPS group d Original cartilage image for Sham group e Original cartilage image for Fracture group f Original cartilage image for PEA-MPS group. (PDF) [file pone.0178553.s003.pdf]

Fig. 4a

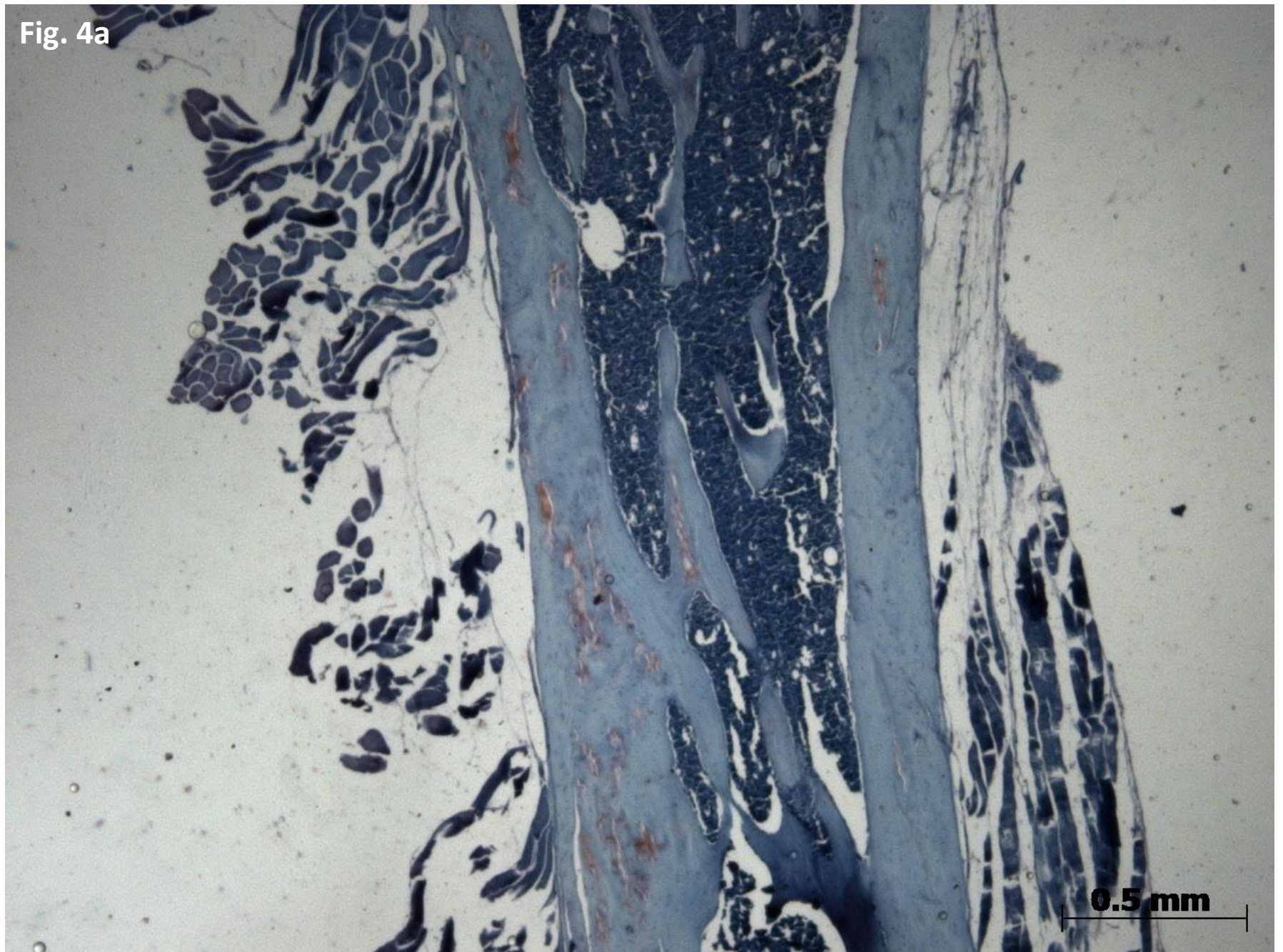

**Fig. 4b**

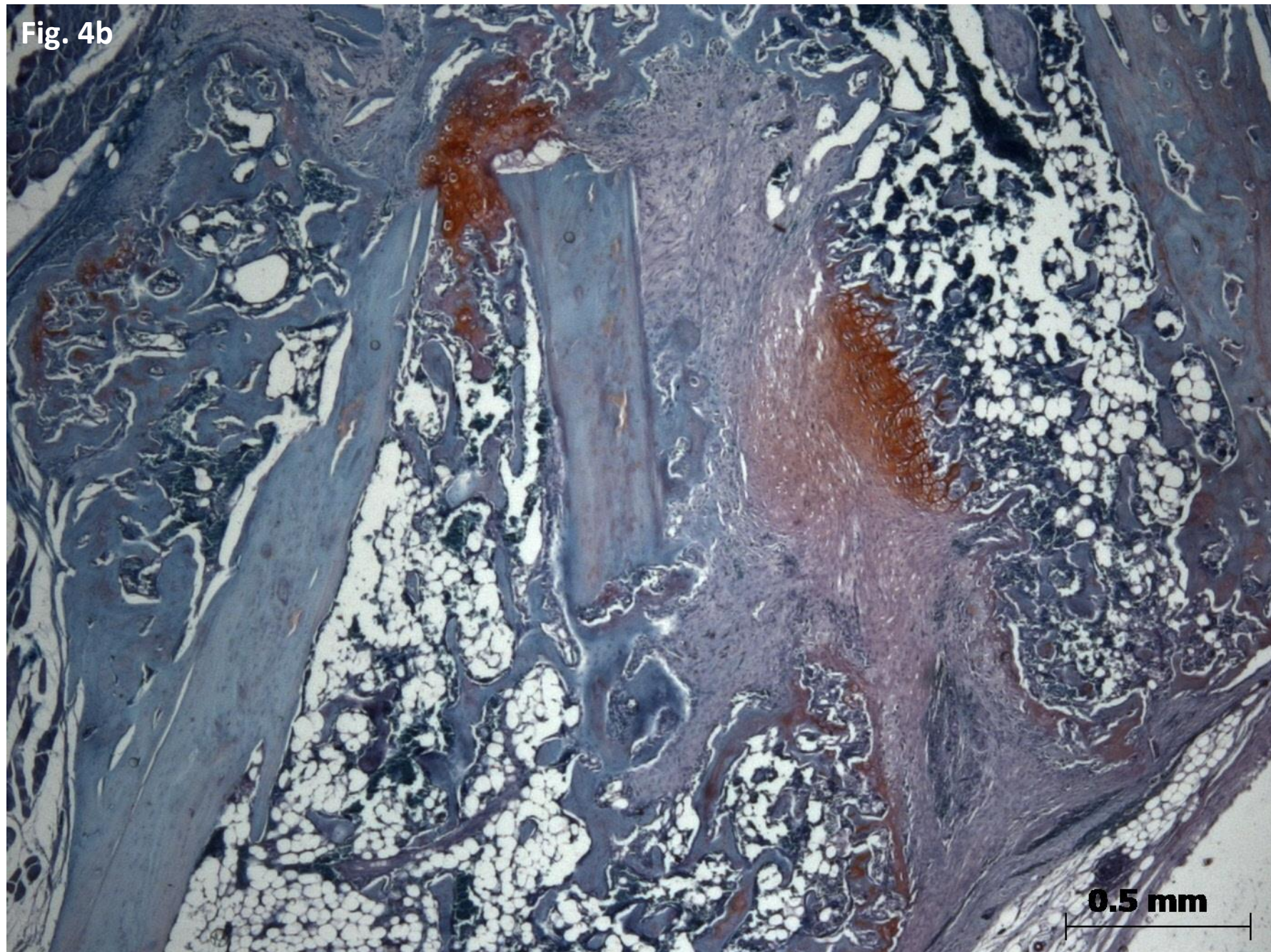

**Fig. 4c**

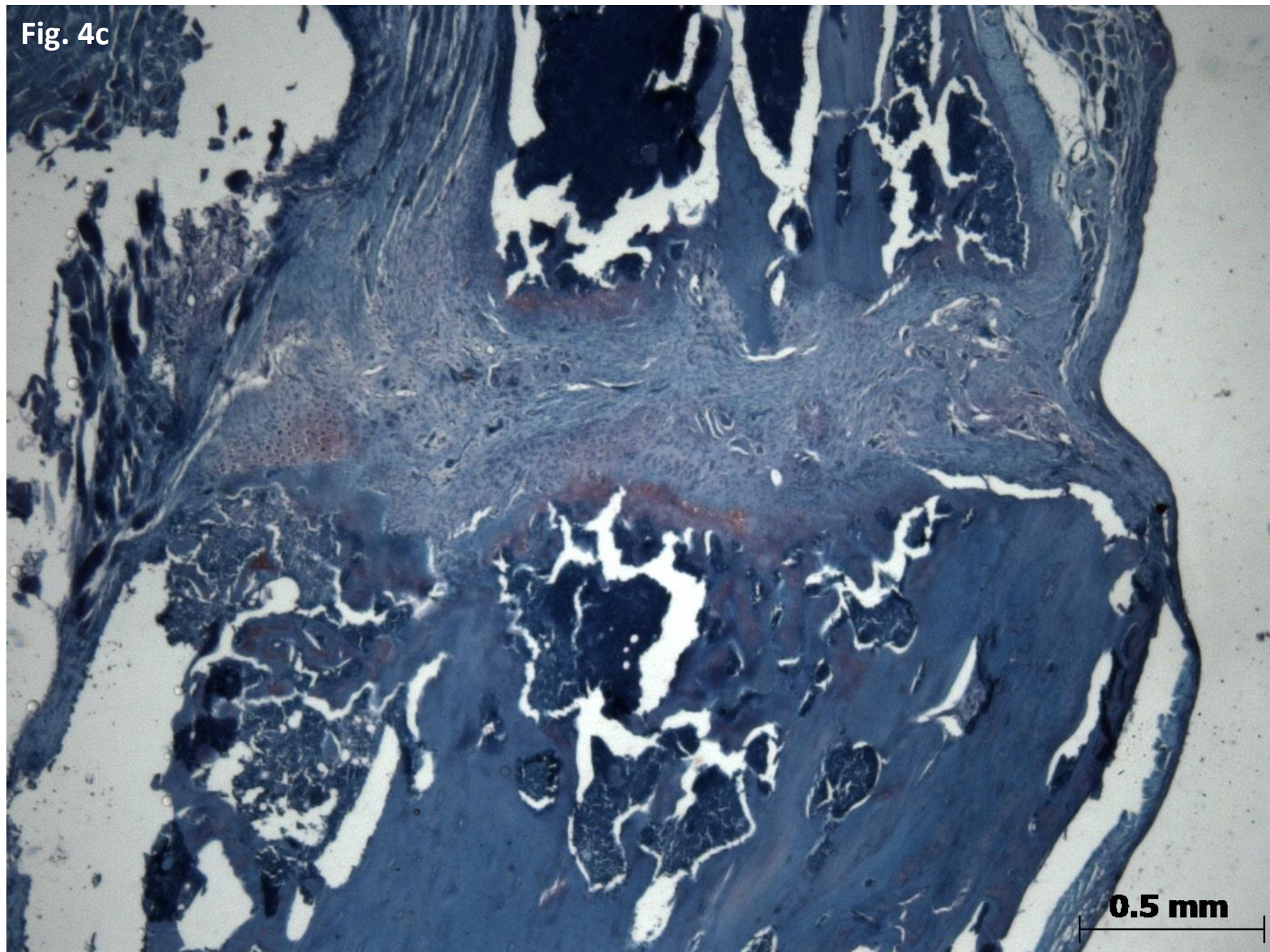

Supplement: S4 File — a Original safranin O/fast green image for osteoclast for Sham group b Original safranin O/fast green image for osteoclast for Fracture group c. Original safranin O/fast green image for osteoclast for PEA-MPS group. (PDF) [file pone.0178553.s004.pdf]

**Fig. 5a**

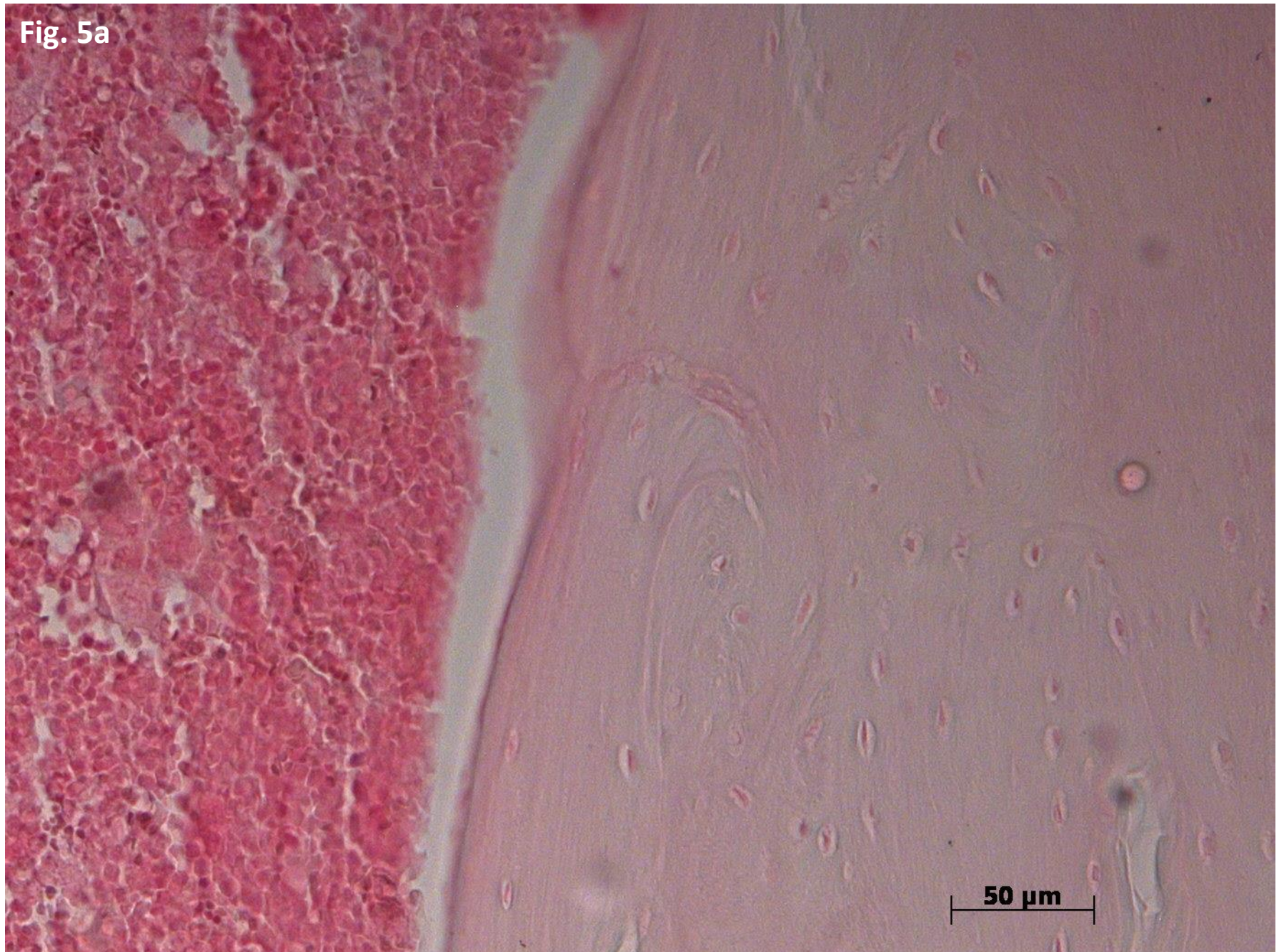

**Fig. 5b**

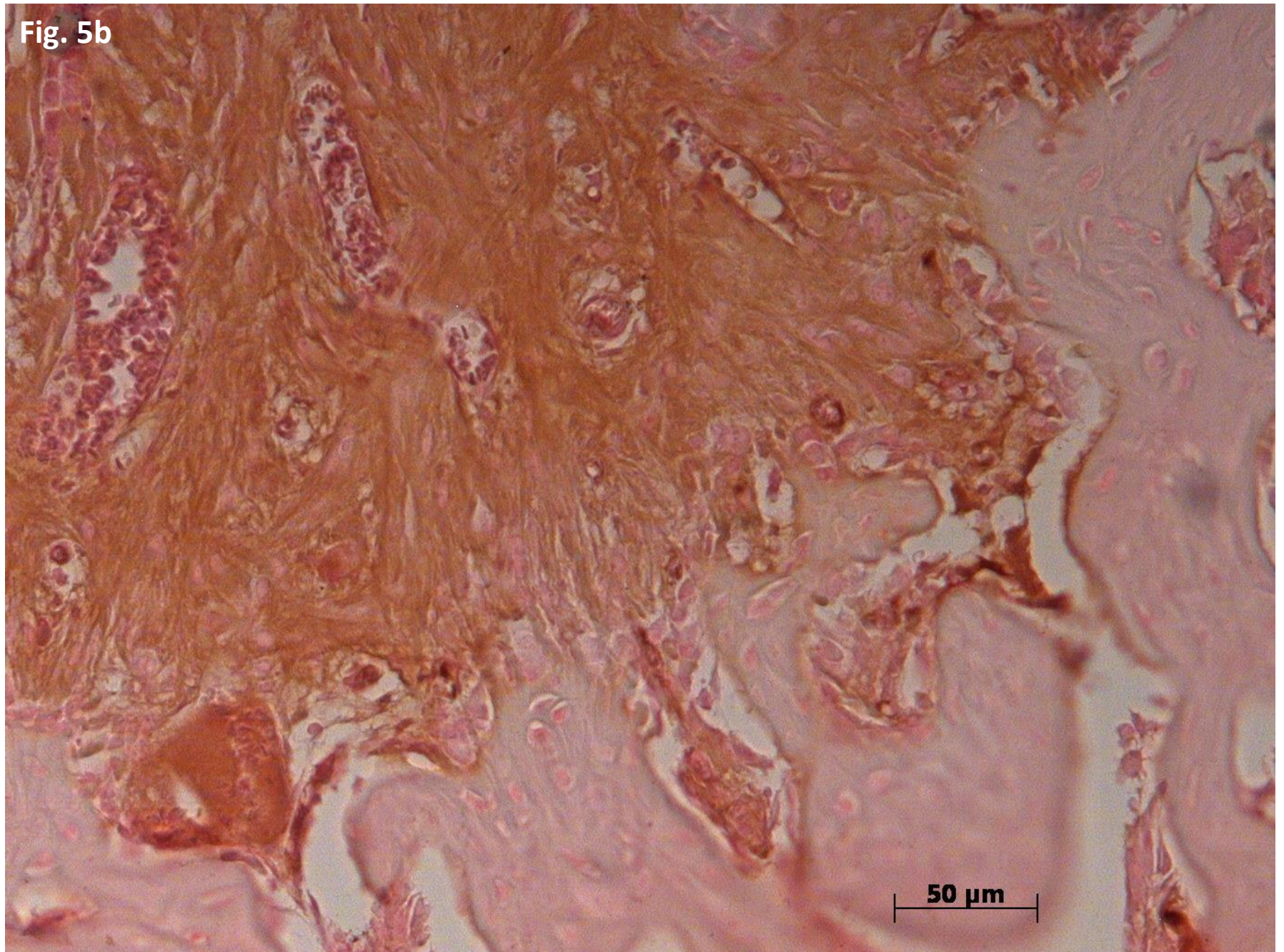

Fig. 5c

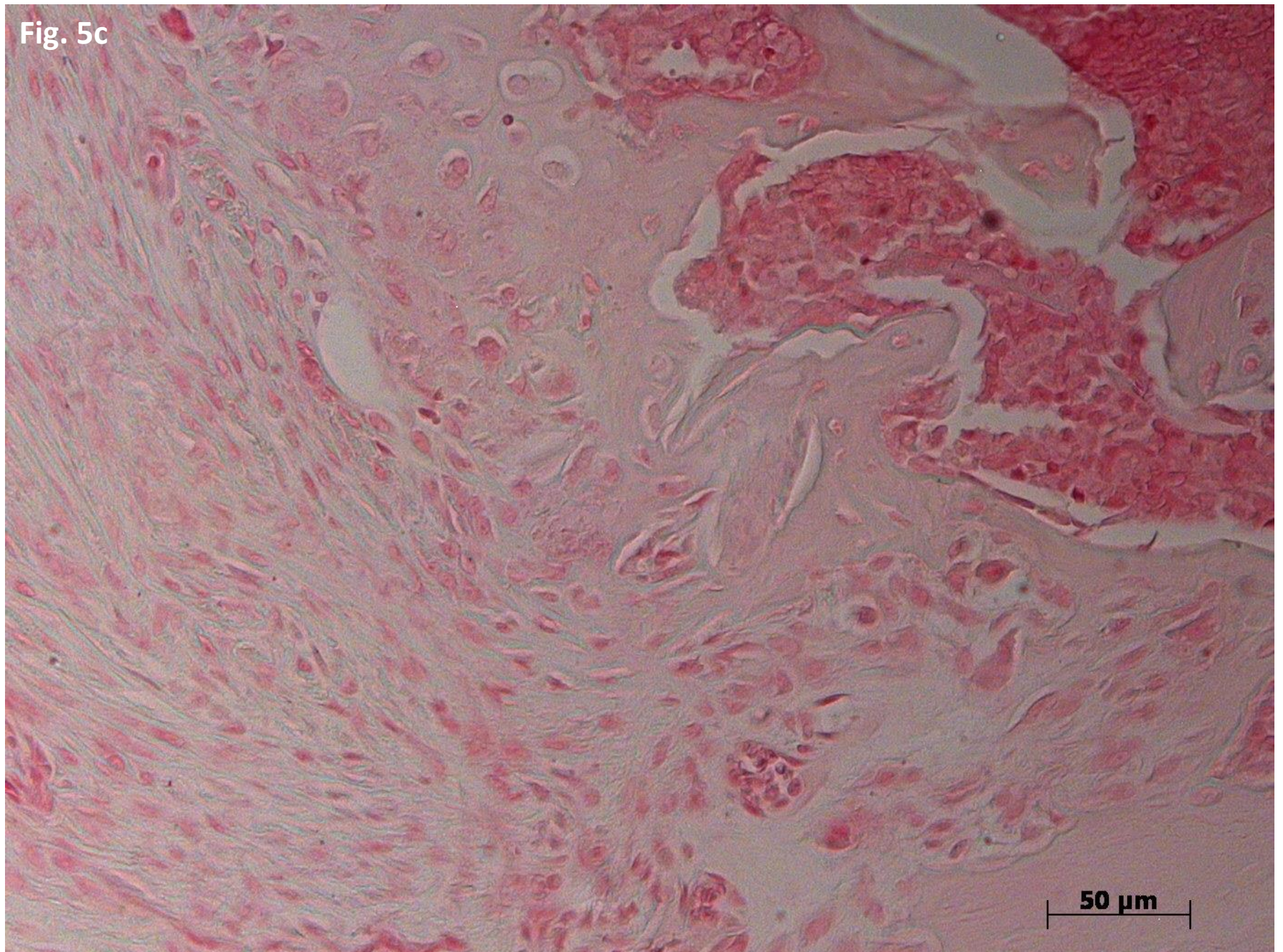

Fig. 5d

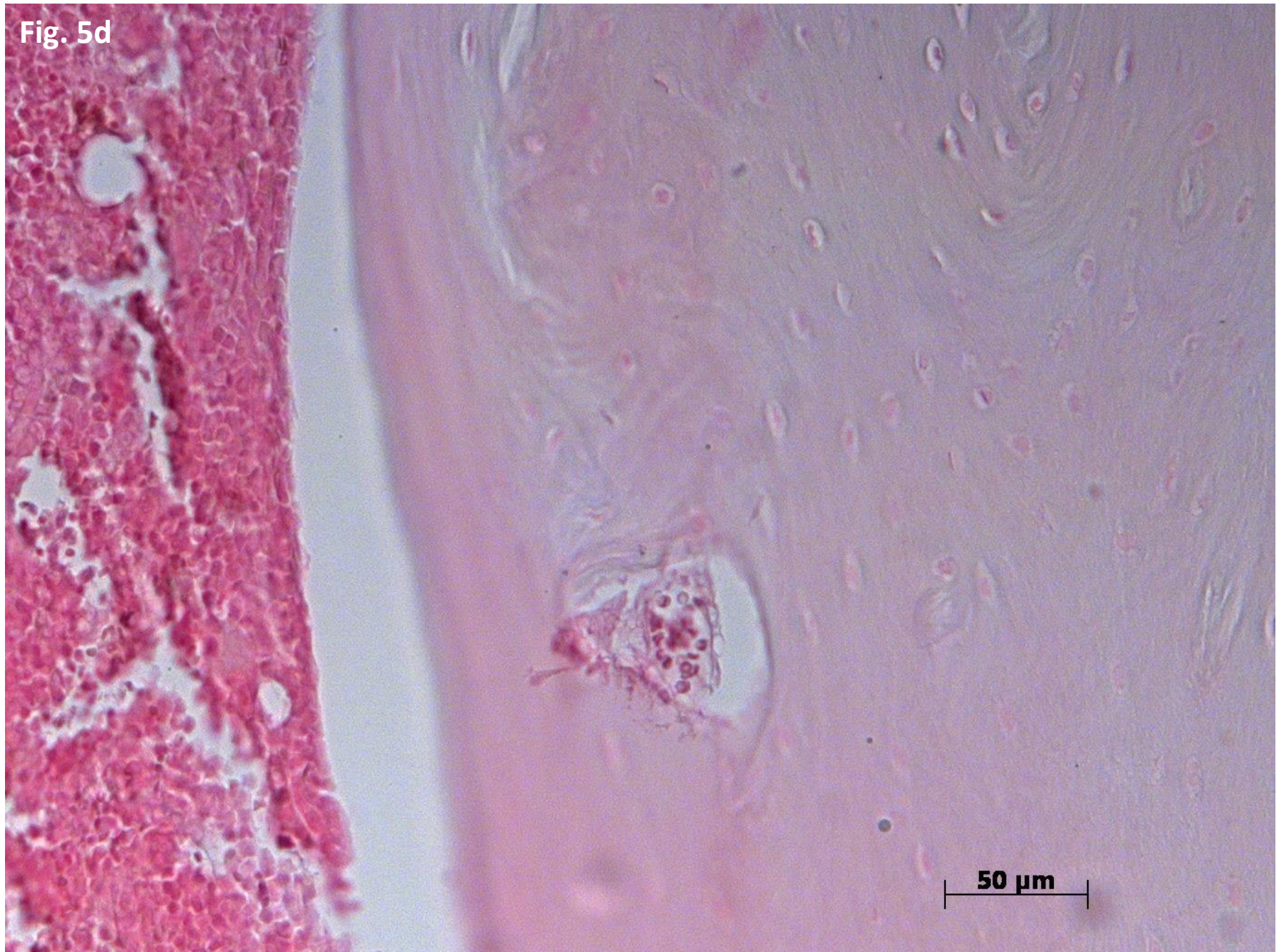

Fig. 5e

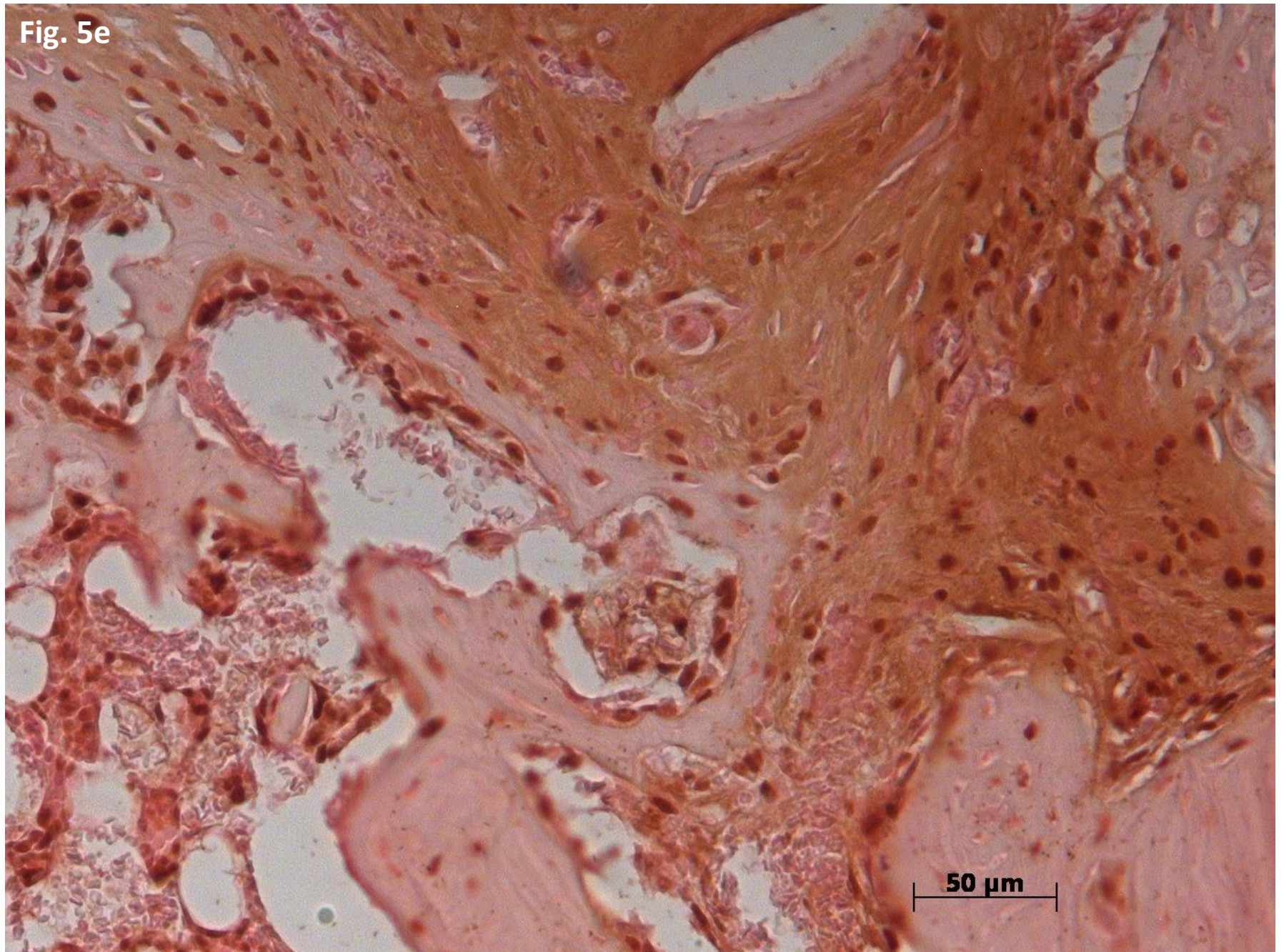

Fig. 5f

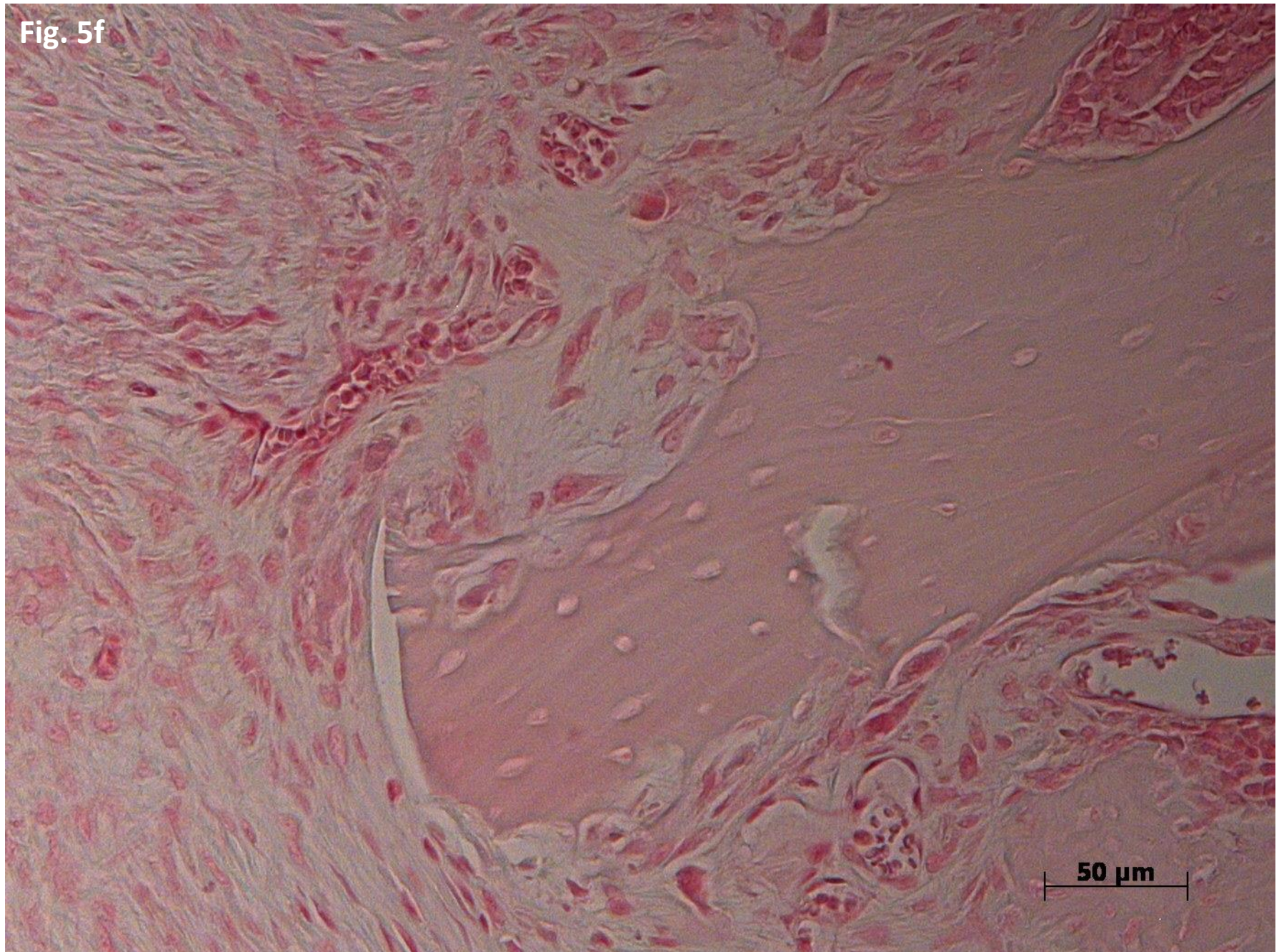

Supplement: S5 File — a Original immunohistochemical image for TNF-α for Sham group b Original immunohistochemical image for TNF-α for Fracture group c. Original immunohistochemical image for TNF-α for PEA-MPS group d Original immunohistochemical image for IL-1β for Sham group e Original immunohistochemical image for IL-1β for Fracture group f Original immunohistochemical image for IL-1β for PEA-MPS group. (PDF) [file pone.0178553.s005.pdf]

Fig. 6a

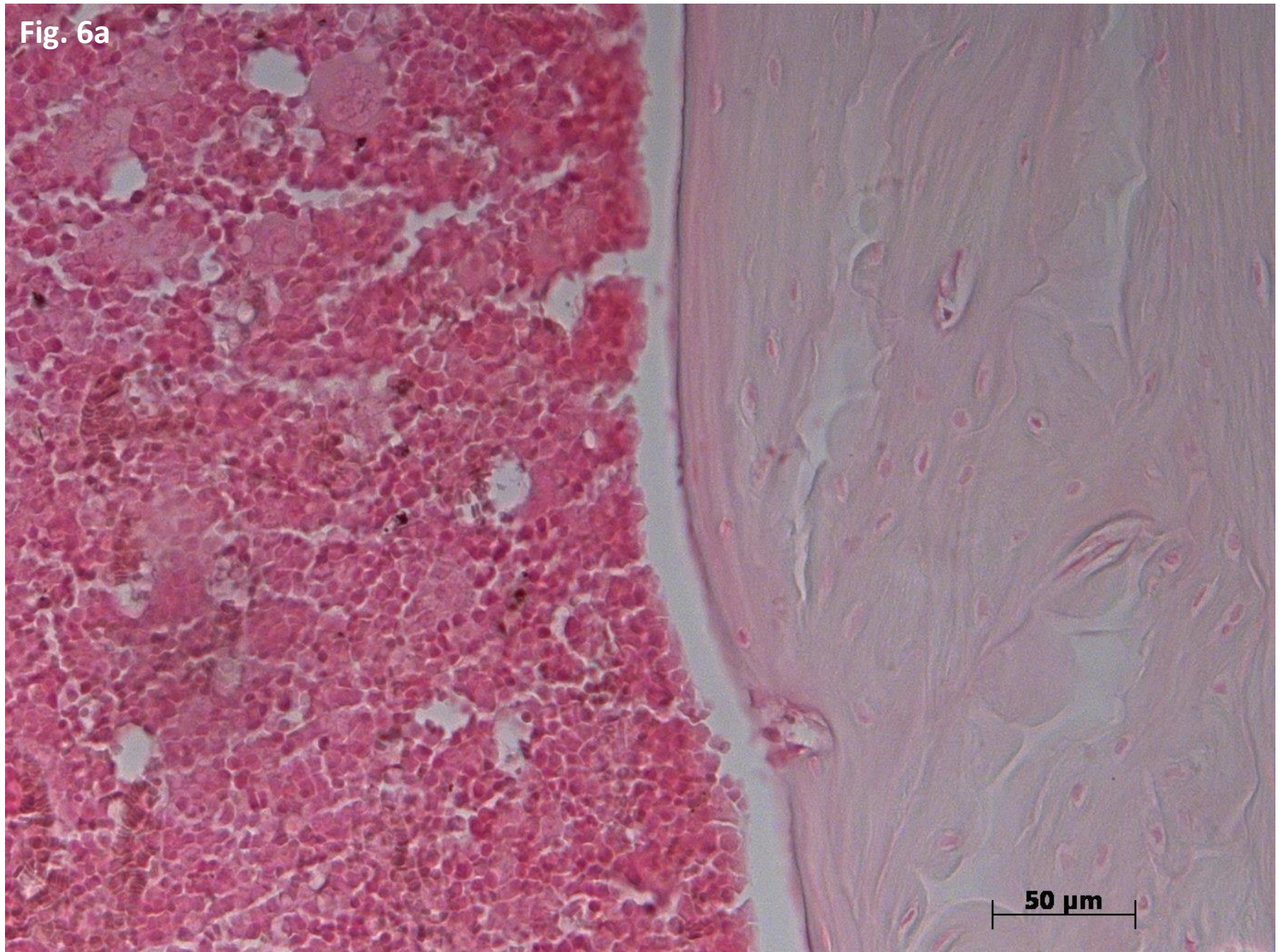

**Fig. 6b**

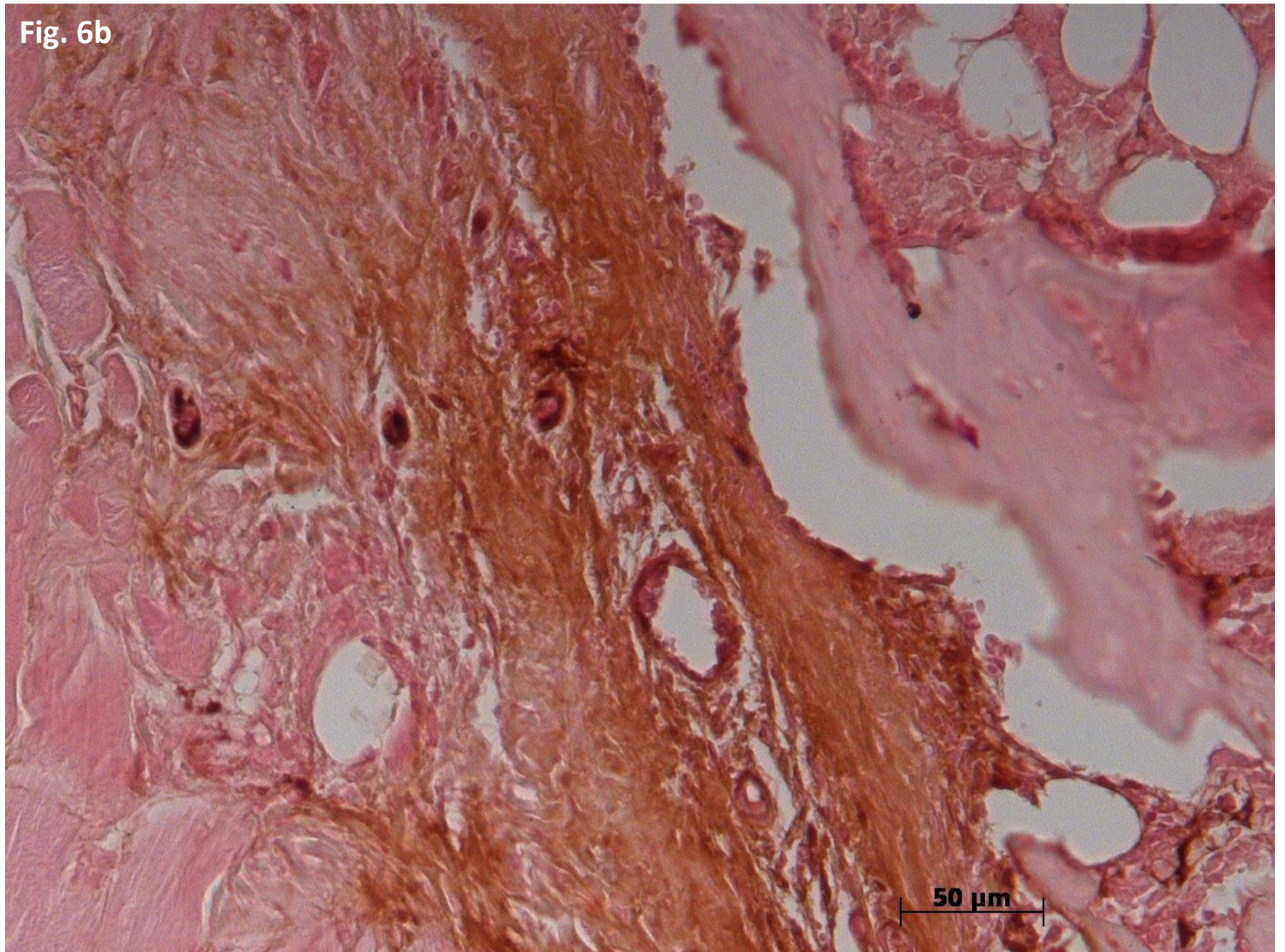

Fig. 6c

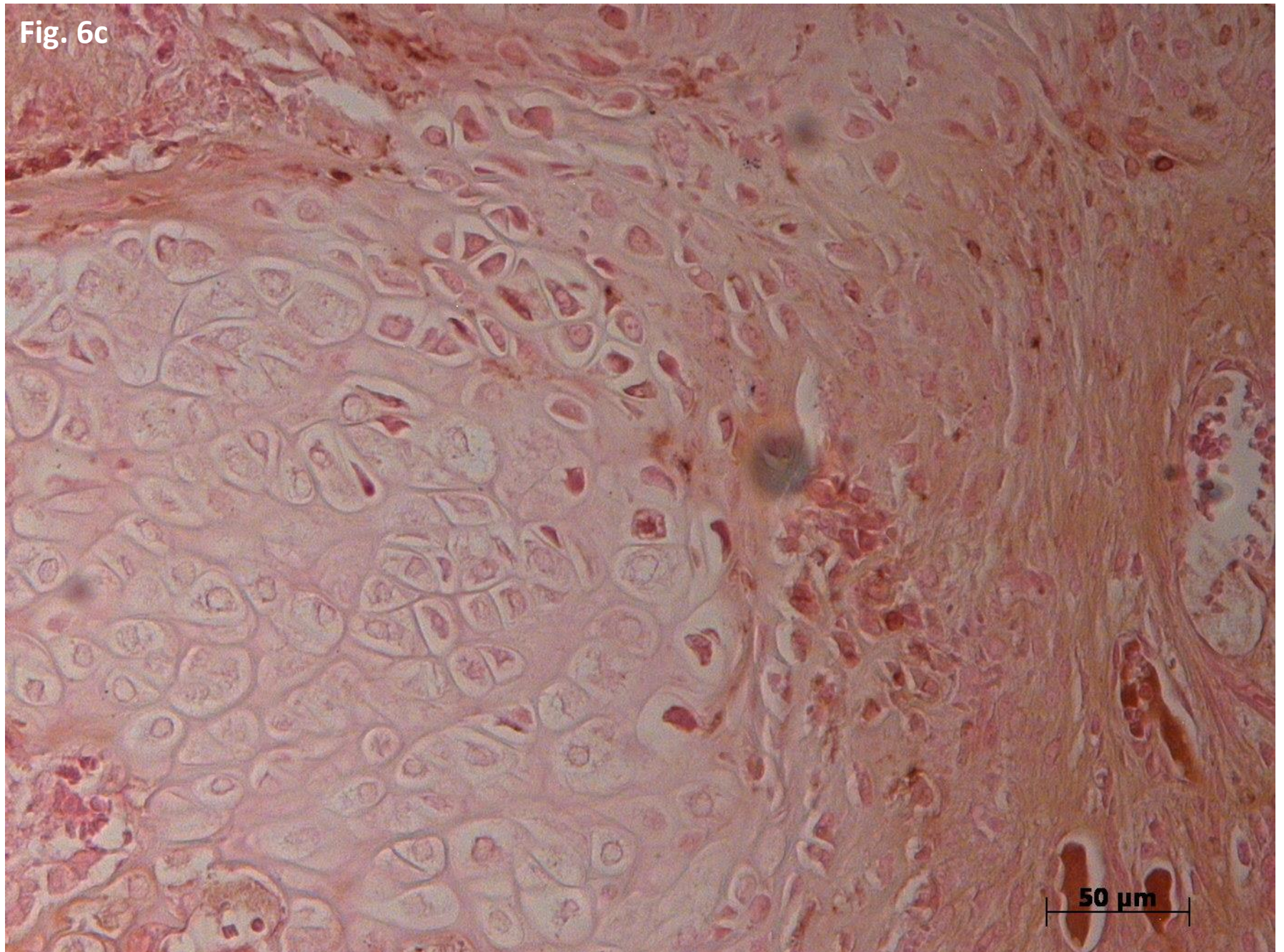

**Fig. 6d**

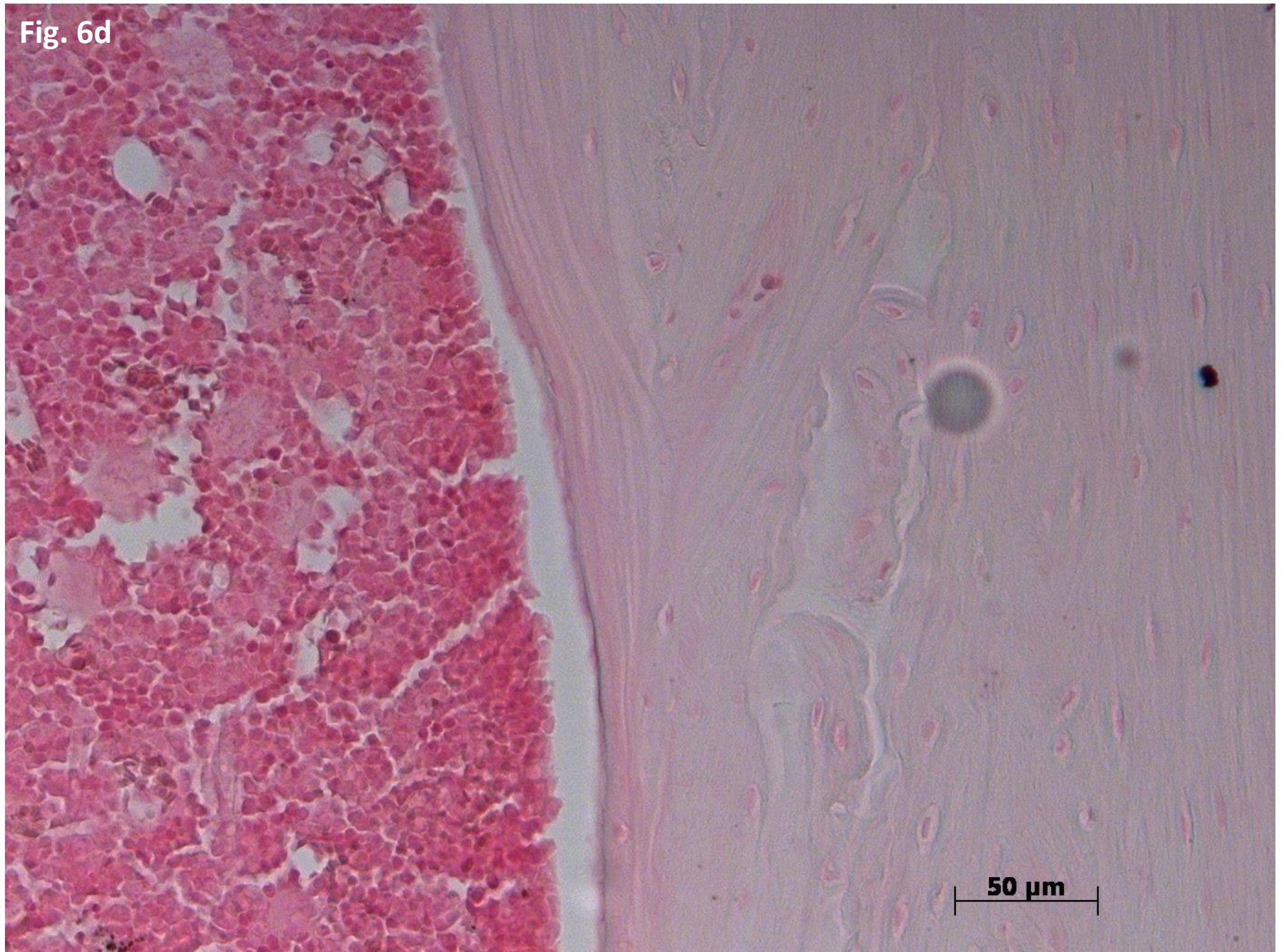

Fig. 6e

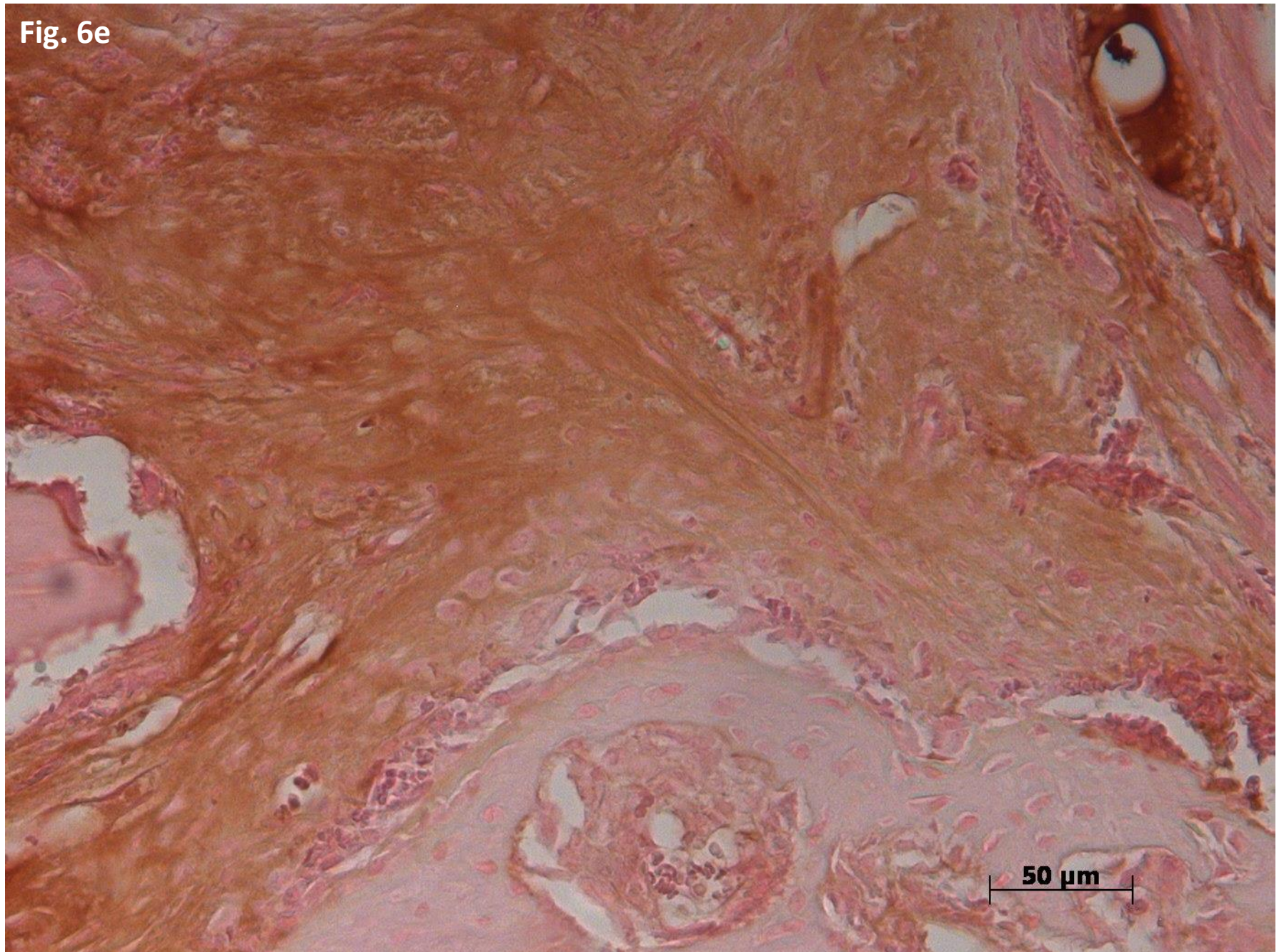

Fig. 6f

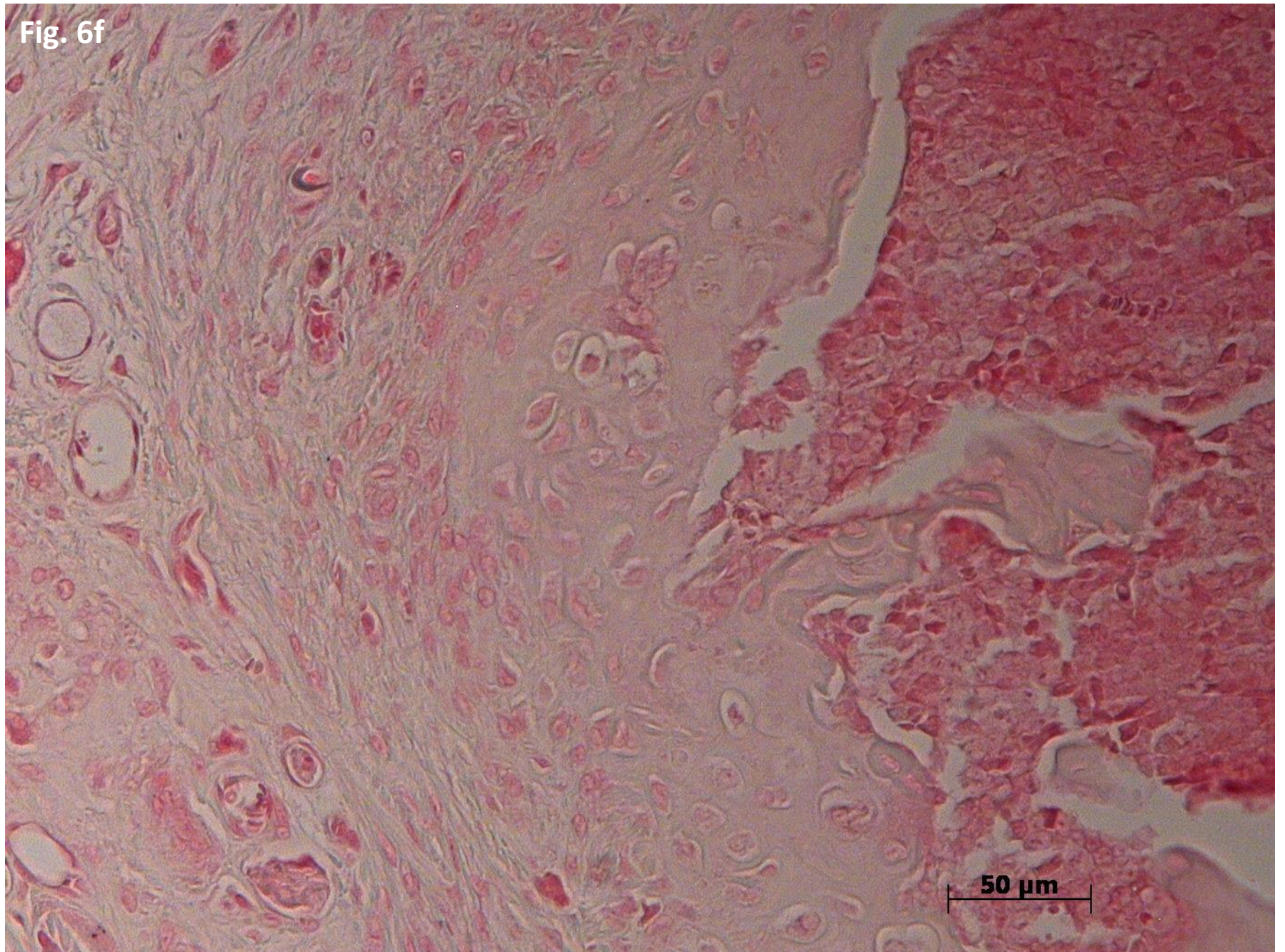

Supplement: S6 File — a Original immunohistochemical image for nitrotyrosine for Sham group b Original immunohistochemical image for nitrotyrosine for Fracture group c. Original immunohistochemical image for nitrotyrosine for PEA-MPS group d Original immunohistochemical image for PAR for Sham group e Original immunohistochemical image for PAR for Fracture group f Original immunohistochemical image for PAR for PEA-MPS group. (PDF) [file pone.0178553.s006.pdf]

**Fig. 7a**

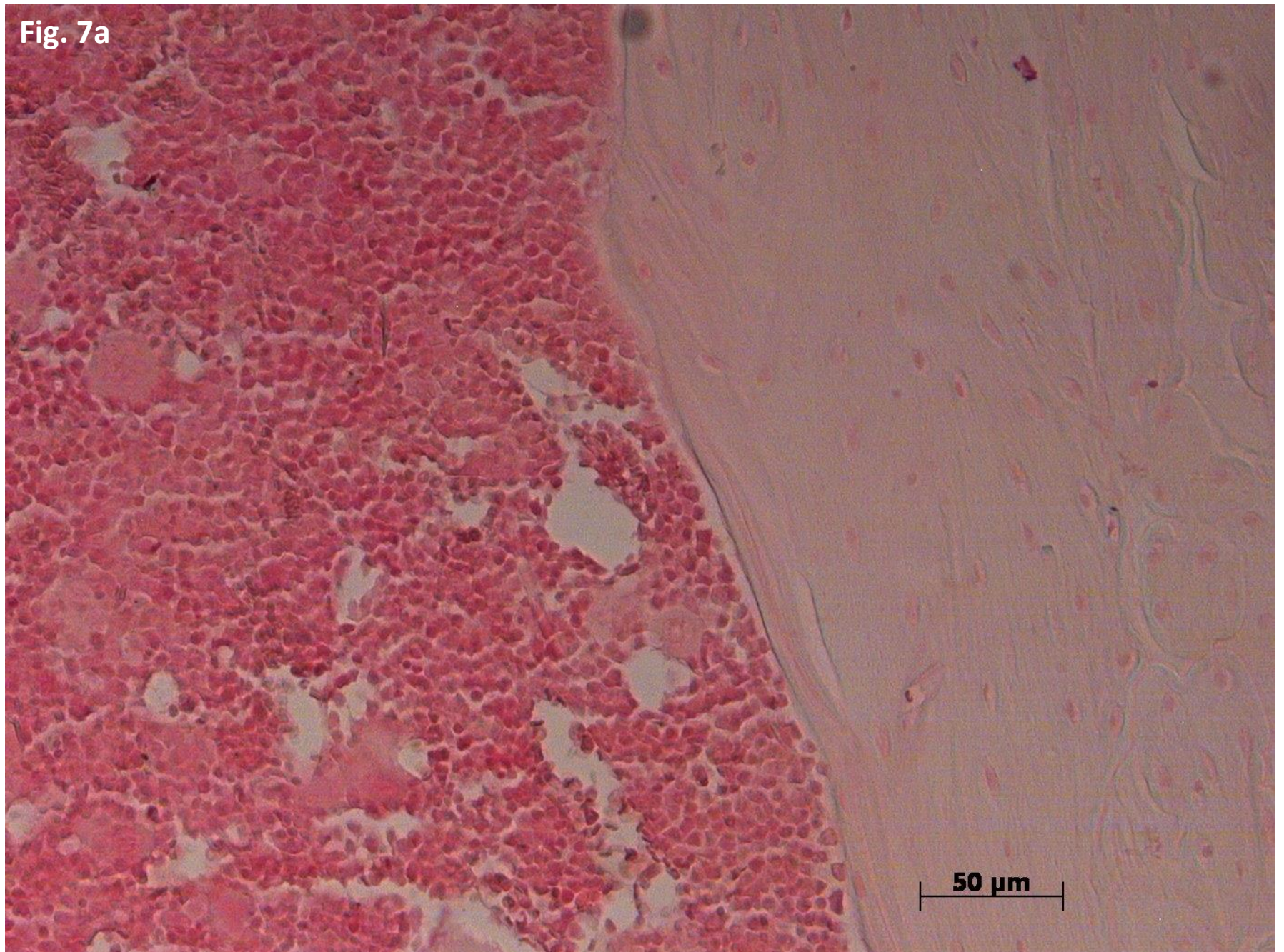

**Fig. 7b**

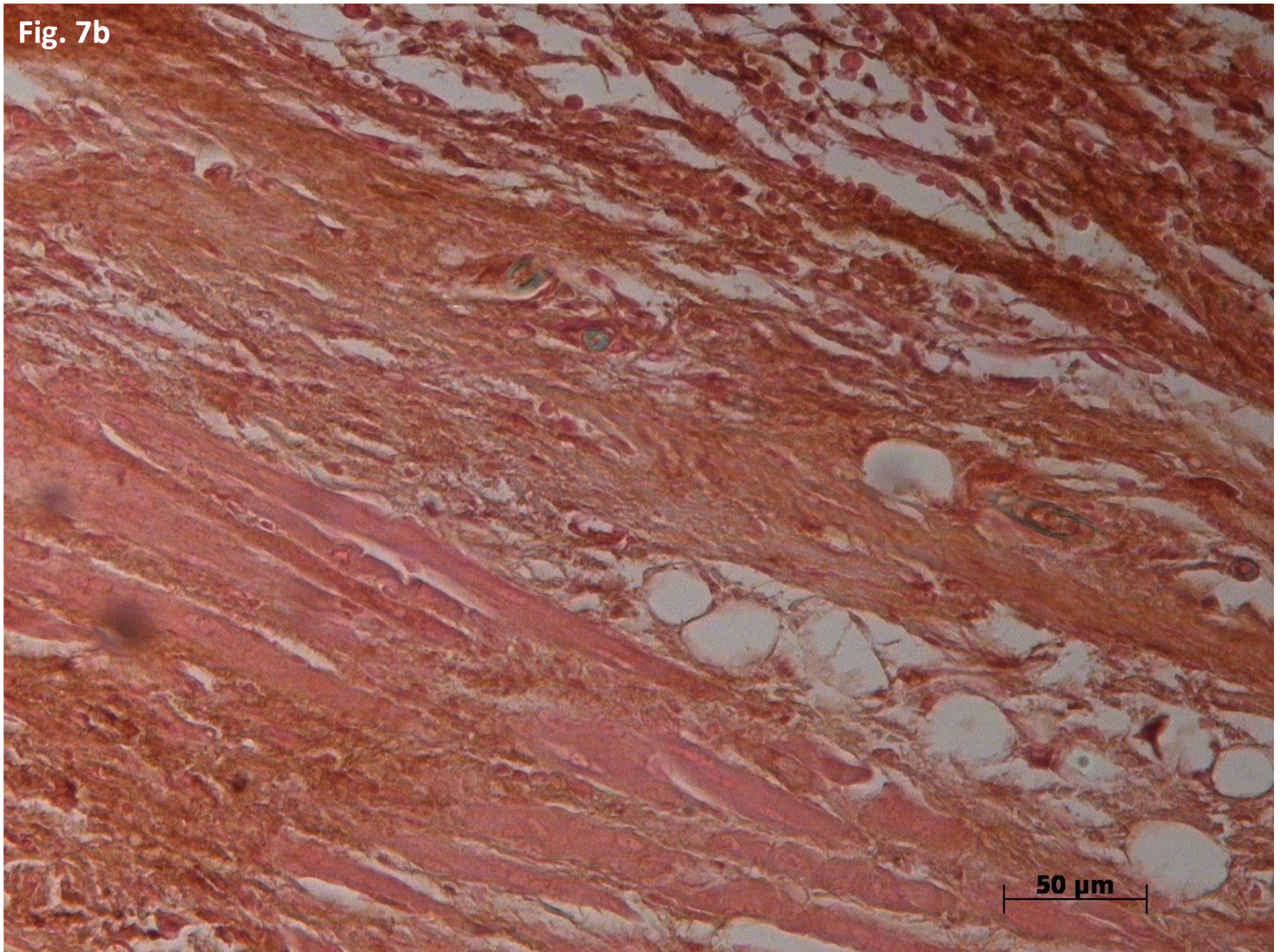

Fig. 7c

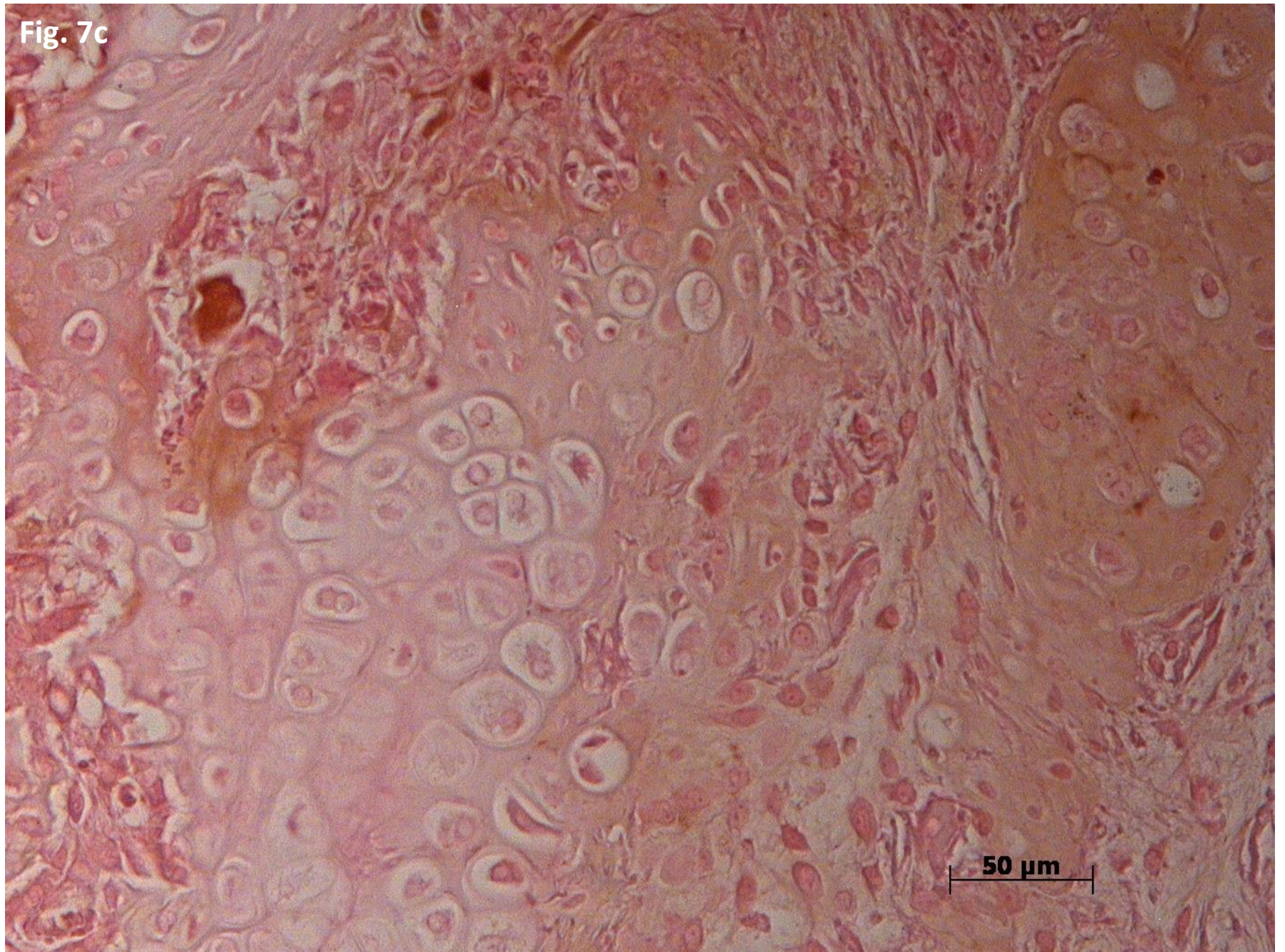

Fig. 7d

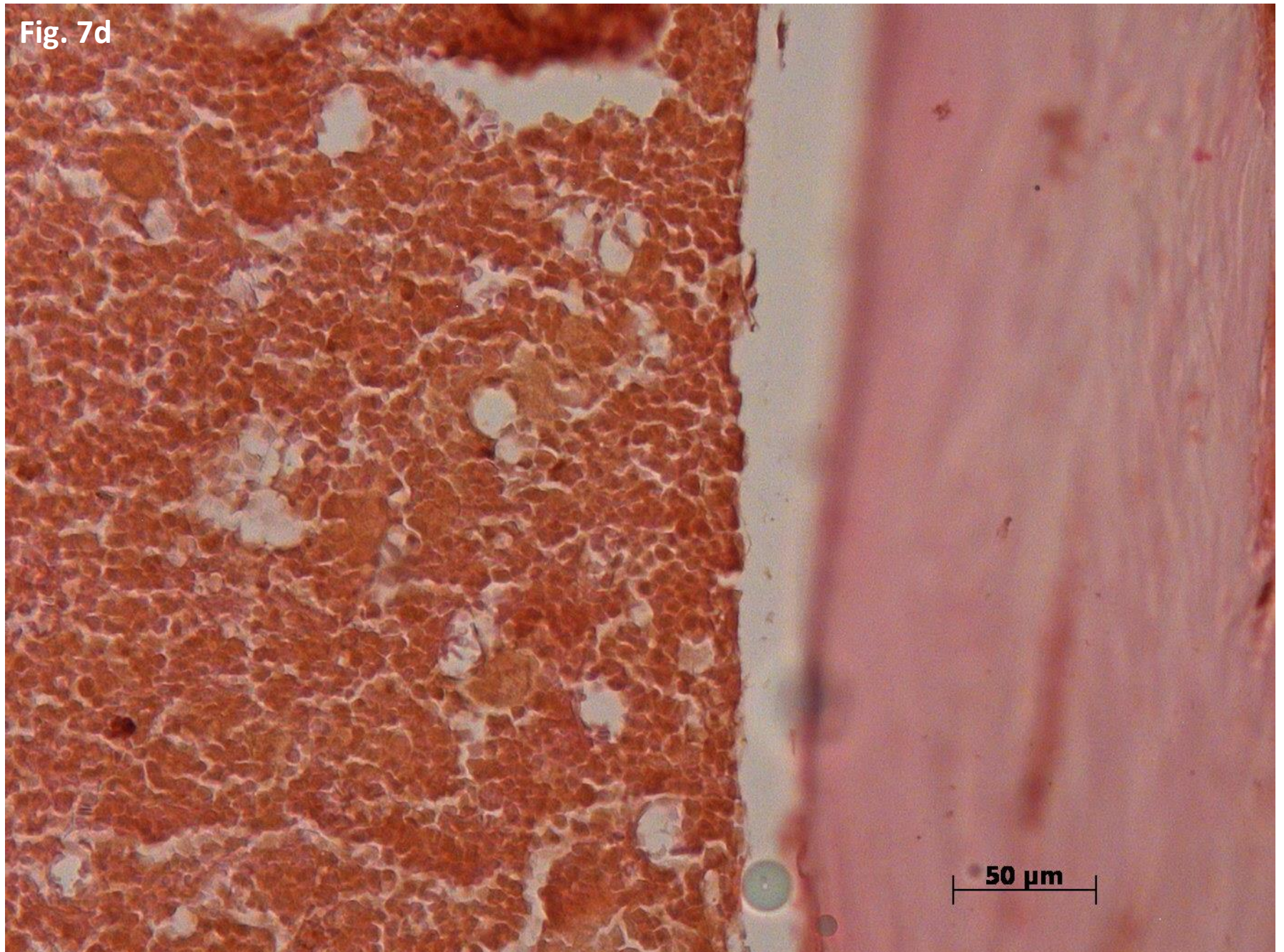

Fig. 7e

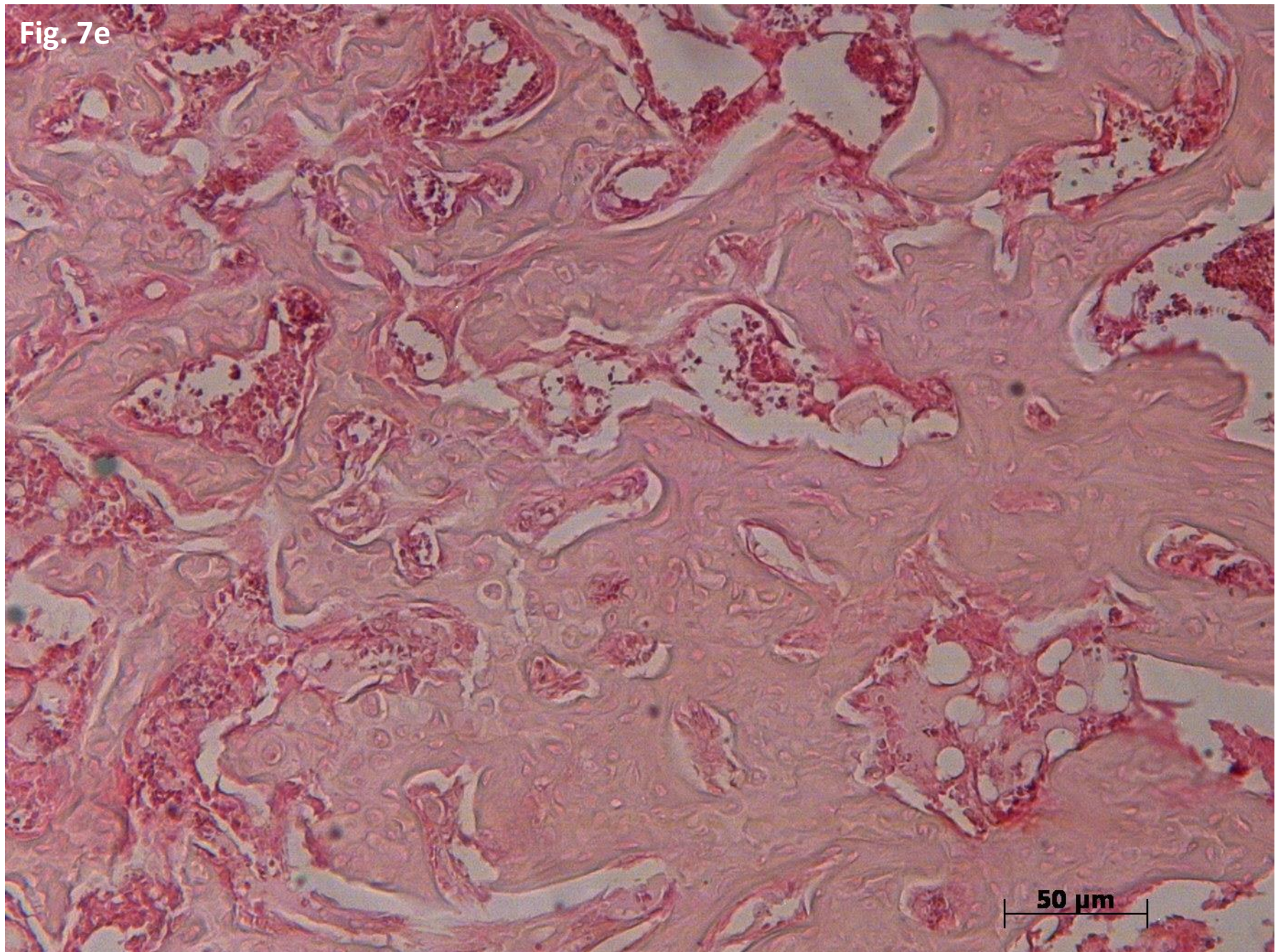

Fig. 7f

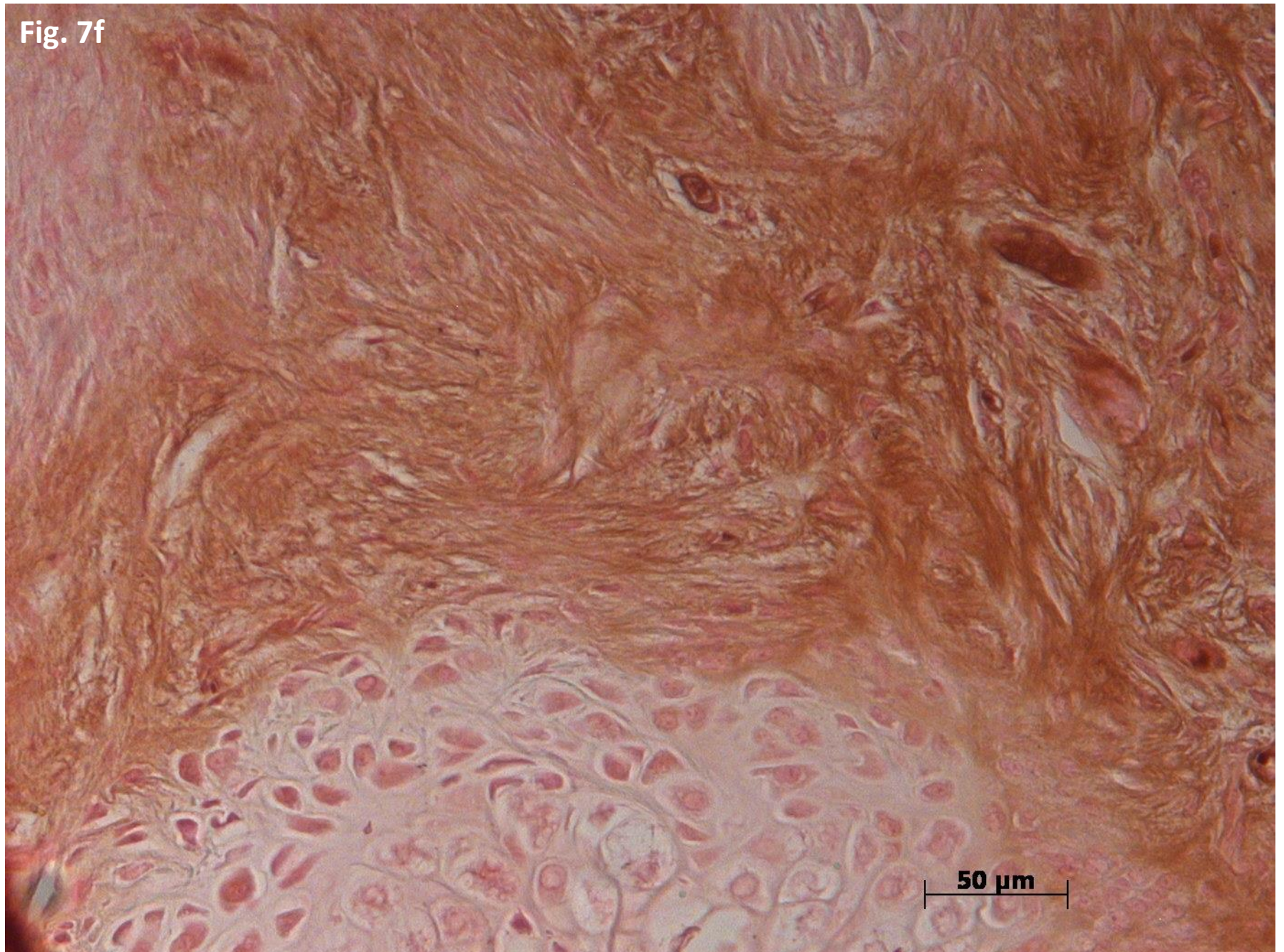

Supplement: S7 File — a Original immunohistochemical image for Bax for Sham group b Original immunohistochemical image for Bax for Fracture group c. Original immunohistochemical image for Bax for PEA-MPS group d Original immunohistochemical image for Bcl-2 for Sham group e Original immunohistochemical image for Bcl-2 for Fracture group f Original immunohistochemical image for Bcl-2 for PEA-MPS group. (PDF) [file pone.0178553.s007.pdf]
